# Taoling Yuyang Decoction alleviates DSS-induced ulcerative colitis in mice by regulating gut microbiota -metabolite axis and restore intestinal barrier function

**DOI:** 10.3389/fmicb.2026.1750380

**Published:** 2026-04-29

**Authors:** Dongyang Liu, Deshuang Lu, Ziwen Zhang, Chang Zhang, Shuang Liu, Jinlai Song, Xingrui Cui, Zhuoyue Dong, Lin Li, Guoying Liang, Chunjing Zhang

**Affiliations:** 1 The First Department of Gastroenterology, The First Affiliated Hospital of Heilongjiang University of Chinese Medicine, Harbin, China; 2 Department of Medical Technology, Qiqihar Medical University, Qiqihar, China; 3 College of Basic Medicine, Heilongjiang University of Chinese Medicine, Harbin, China; 4 Jiangsu Hospital with Integration of Chinese and Western Medicine, Nanjing University of Chinese Medicine, Nanjing, China

**Keywords:** antioxidant function, gut microbiota, intestinal barrier, Taoling Yuyang Decoction, ulcerative colitis

## Abstract

**Background:**

Traditional Chinese medicine (TCM) offers multi-targeted therapy with few side effects for ulcerative colitis (UC). Taoling Yuyang Decoction (TLYY) has shown clinical efficacy, however, its mechanisms of action are unclear. This study employed a multi-omics approach to elucidate TLYY’s therapeutic effects in a murine DSS-induced colitis model.

**Methods:**

Using a DSS-induced mouse model of colitis, animals were divided into five groups (*n* = 8): blank control (CK), model (DSS), low- and high-dose TLYY (TLYYL, TLYYH), and positive control (5-ASA). Treatment efficacy was assessed by measuring body weight, disease activity index (DAI), and histopathological changes. Oxidative stress markers (MDA, GSH-Px, SOD) in serum and colon tissues were quantified using commercial kits and immunohistochemistry. Cytokine levels (IL-1β, TNF-α, IL-6) and LPS were measured by ELISA. The expression of tight junction proteins (ZO-1, Occludin, Claudin-1) in colon tissue was analyzed via qPCR and immunocytochemistry. Additionally, 16S rDNA sequencing and metabolomics were employed to examine gut microbiota composition, fecal SCFAs, and differential metabolites.

**Results:**

Taoling Yuyang Decoction treatment dose-dependently alleviated UC symptoms, including weight loss, colon shortening, and tissue damage. It enhanced antioxidant activities (SOD, GSH-Px) and reduced MDA, suppressed pro-inflammatory cytokines and LPS, and up-regulated tight junction proteins (ZO-1, Occludin, Claudin-1). Gut microbiota analysis showed TLYY-H restored microbial diversity, increased beneficial bacteria (e.g., *Lactobacillus*, *Akkermansia*), and decreased harmful genera (e.g., *Helicobacter*). It also elevated SCFA levels and modulated fecal metabolism, up-regulating anti-inflammatory/antioxidant metabolites while down-regulating toxic compounds. Key pathways such as AGE-RAGE signaling and xenobiotic metabolism were restored.

**Conclusion:**

Taoling Yuyang Decoction ameliorates DSS-induced colitis via integrated mechanisms involving oxidative stress reduction, anti-inflammation, intestinal barrier repair, and restoration of gut flora homeostasis and metabolic pathways. The superior effect of the high dose highlights its clinical potential for UC treatment.

## Introduction

Ulcerative colitis (UC) is a lifelong, complex, chronic, immune-mediated inflammatory disease of the colon. Its primary clinical manifestations include abdominal pain, diarrhea, mucopurulent bloody stools, and tenesmus (Sun et al., 2026; Lv et al., 2025). The pathogenesis of UC is closely associated with intestinal dysbiosis, impairment of the intestinal barrier, and immune dysregulation (Nascimento et al., 2020; Jiang and Miao, 2023). Conventional medical treatment primarily relies on immunosuppressants, but long-term use can lead to severe adverse effects. In contrast, Traditional Chinese Medicine (TCM) demonstrates unique advantages through its multi-target approach, holistic regulation, and minimal side effects (Zhang et al., 2024; Chen et al., 2025). Recent studies have revealed that UC patients commonly exhibit gut microbiota dysbiosis, characterized by a reduction in beneficial bacteria, overgrowth of pathogenic bacteria, and insufficient production of short-chain fatty acids (SCFAs). This microecological imbalance not only exacerbates intestinal mucosal inflammation but also disrupts the intestinal mucosal barrier through toxins produced by dysbiotic microbiota (Mirsepasi-Lauridsen et al., 2022; Cheng et al., 2025). TCM compound formulations, such as Baitouweng Decoction (Pan et al., 2024), extracts of Painong San (Wang et al., 2022) and Gancao Xiexin Decoction (Pan et al., 2025) exert therapeutic effects through multiple pathways, including modulating microbiota composition, inhibiting inflammatory signaling pathways, and enhancing intestinal barrier function.

In-depth research indicates that the core mechanism of TCM in treating UC lies in regulating the “microbiota-immune-barrier” axis. Regarding microbiota modulation, active components of Chinese herbs can reduce the abundance of Firmicutes and the Firmicutes/Bacteroidetes (F/B) ratio, while increasing the relative abundance of SCFA-producing bacteria, such as those generating propionic and butyric acid, thereby restoring microecological balance (Zhang Y. et al., 2025). At the immune regulation level, TCM compounds, by modulating the gut microbiota, decrease the levels of pro-inflammatory cytokines like TNF-α and IL-6, and regulate the Th17/Treg cell balance (Peng et al., 2024; Huang et al., 2025). For intestinal barrier repair, TCM compounds and active substances not only upregulate the expression of tight junction proteins but also enhance the chemical barrier by promoting mucin secretion and providing energy for colonic epithelial cells via increased SCFA production. Notably, excessive reactive oxygen species (ROS) in the intestines of UC patients can directly damage epithelial cells, and TCM components, such as those in Ganjiang Huangqin Huanglian Renshen Decoction, can alleviate mitochondrial oxidative stress injury in DSS-induced ulcerative colitis (Zhou et al., 2024). This multi-target synergistic mode of action represents the characteristic and advantage of TCM in treating UC.

Based on the primary etiology, pathogenesis, and typical clinical manifestations of UC, and in accordance with the fundamental theories of TCM and the principle of treatment based on syndrome differentiation, our research group developed Taoling Yuyang Decoction (TLYY) for the treatment of UC with the Dampness Stagnancy due to Spleen Deficiency. TLYY is formulated based on the therapeutic principle of invigorating the spleen and replenishing qi, as well as relieving diarrhea and eliminating dampness. It is composed of the following herbs: *Halloysitum Rubrum*, *Rhizoma Zingiberis*, *Codonopsis pilosula (Franch.) Nannf.*, *Radix Astragali*, *Atractylodes macrocephala Koidz.*, *Dioscorea opposita*, *Pulsatilla chinensis Regel*, *Portulaca oleracea L.*, *Coptis chinensis Franch*, and *Atractylodes Lancea (Thunb.) DC*. In this formula, *Codonopsis pilosula (Franch.) Nannf.* invigorates spleen and replenishes qi, *Radix Astragali* benefits qi and harmonizes middle energizer, and *Atractylodes macrocephala Koidz* invigorates spleen and replenishes qi to eliminate dampness. These three herbs serve as the *Sovereign(Jun)* drugs, working synergistically to invigorate spleen and replenish qi to harmonize the middle energizer. *Coptis chinensis Franch* clears heat and promotes diuresis, and *Atractylodes Lancea (Thunb.) DC*. invigorates spleen to eliminate dampness; these two serve as the Minister (Chen) drugs. Dioscorea opposita invigorates spleen and kidney; *Pulsatilla chinensis Regel* and *Portulaca oleracea L.* clear the intestines and cure dysentery and cool blood achieve hemostasis. *Halloysitum Rubrum* relieves diarrhea with astringents, and *Rhizoma Zingiberis* warms spleen and stomach to dispel cold; together, these two herbs achieve the effects of the warmly invigorating spleen for hemostasis and relieving diarrhea with astringents. The combination of all herbs works synergistically to invigorate the spleen, eliminate dampness, and relieve diarrhea with astringents. Preliminary clinical results from our group indicated that UC patients receiving oral TLYY experienced effective alleviation of TCM clinical symptoms associated with the Dampness Stagnancy due to Spleen Deficiency (Li, 2023). Furthermore, significant reductions in levels of C-reactive protein (CRP) and erythrocyte sedimentation rate (ESR) were observed. However, its underlying mechanism of action remains unclear. This study intends to further investigate TLYY as the therapeutic intervention. We plan to establish a dextran sulfate sodium (DSS)-induced ulcerative colitis mouse model to evaluate the efficacy of TLYY in alleviating DSS-induced colitis and to explore its potential mechanism of action. This will be achieved by analyzing changes in the intestinal microbial community structure and host metabolite profiles in the model mice. The aim is to elucidate the mechanism by which TLYY alleviates UC, thereby providing a theoretical basis for the use of TLYY in treating UC and offering new insights and methodologies for TCM-based UC treatment.

## Materials and methods

### Materials and reagents

Taoling Yuyang Decoction (TLYY) was prepared by mixing the following crude herbs in the specified ratios: *Halloysitum rubrum*, *Rhizoma Zingiberis*, *Codonopsis pilosula (Franch.) Nannf.*, *Radix Astragali*, *Atractylodes macrocephala Koidz.*, *Dioscorea opposita Thunb.*, *Pulsatilla chinensis (Bunge) Regel*, *Portulaca oleracea L.*, *Coptis chinensis Franch.*, and *Atractylodes lancea (Thunb.)* DC, at a weight ratio of 1.5:1:2:2:1.5:1.5:1.5:2:2:1.

The herbal mixture was boiled in 8 volumes (v/w) of water for 1 h and filtered while hot. The residue was boiled again in 6 volumes (v/w) of water for another hour. The two filtrates were combined and concentrated to a final concentration of 1 g/mL (crude drug weight/volume). Metabolite analysis of the TLYY extract was performed by Shanghai Personal Biotechnology Co., Ltd., (Shanghai, China). A total of 3,114 metabolites were detected in the concentrated extract. The majority of these belonged to the following classes: carboxylic acids and derivatives, prenol lipids, benzene and substituted derivatives, and fatty acyls. The top 50 metabolites, ranked by peak area, are listed in Supplementary Table 1.

For the 5-ASA suspension, 0.5% carboxymethyl cellulose sodium (CMC-Na) was used as a suspending agent to prepare a final concentration of 0.025 g/mL. Accurately weigh the 5-ASA powder (Shanghai Aidefa Pharmaceutical Co., Ltd., Approval no. H20040727), add an appropriate amount of CMC-Na solution, and perform initial homogenization by vortexing. Subsequently, ultrasonic treatment was applied to ensure thorough dispersion and uniformity of the drug particles. The suspension was then adjusted to the final volume and stored at 4 °C for later use. Prior to administration, the suspension was vortexed again to ensure homogeneity and stability.

### Animal models and treatment

Forty male C57BL/6J mice (20 ± 2 g) were provided by the Experimental Animal Center of Qiqihar Medical University [License No. SYXK (HEI) 2021-013]. The mice were housed under standard specific pathogen-free (SPF) conditions: temperature 22 °C–24 °C, relative humidity 60%–65%, and a 12/12-h light/dark cycle, with free access to food and water. All animal experiments were approved by the Animal Ethics Committee of Qiqihar Medical University (Approval No.: 2022401).

After a 7-days acclimatization period, the mice were randomly assigned to five groups (*n* = 8 per group), Normal Control (NC) group: did not undergo modeling induction; administered an equal volume of drinking water by gavage. DSS-induced model (DSS) group, which underwent DSS modeling and received an equal volume of drinking water by gavage. TLYY low-dose (TLYYL) group: Subjected to DSS modeling; administered TLYY at 2 g/kg/day by gavage. TLYY high-dose (TLYYH) group: subjected to DSS modeling; administered TLYY at 4 g/kg/day by gavage. Mesalazine positive control (5-ASA) group: subjected to DSS modeling; administered mesalazine (5-ASA) at 100 mg/kg/day by gavage. To induce colitis, all groups except the NC group received 3.0% (w/v) dextran sulfate sodium (DSS) dissolved in sterile drinking water *ad libitum* for 7 days, while the NC group received sterile drinking water only. This was followed by a 3-days recovery period during which all mice received regular sterile water *ad libitum*. Body weight was recorded daily. Stool consistency and fecal occult blood were monitored to calculate the Disease Activity Index (DAI) according to a previously reported scoring system (Yang et al., 2025). The DAI was used to evaluate the success of the model and the therapeutic efficacy of TLYY.

On day 9 of the experiment, fecal samples were collected and stored at −80 °C for subsequent analysis of the gut microbiota and short-chain fatty acids (SCFAs). On the final day, following a 12-h fast (with water provided *ad libitum*), mice were anesthetized with 3% isoflurane. Upon the loss of the righting reflex (typically within 2–5 min), blood samples were collected via the retro-orbital plexus, and the mice were euthanized by cervical dislocation. Subsequently, tissue samples were collected, and the colon length was measured. Segments of the distal colon were fixed in 4% paraformaldehyde for over 24 h at room temperature in preparation for hematoxylin and eosin (H&E) staining and immunohistochemical analysis. The remaining tissue samples were stored at −80 °C for further analysis.

### Determination of serum and colon tissue oxidative stress and inflammatory cytokine levels

The levels of inflammatory cytokines (TNF-α, IL-1β, and IL-6) in colon tissue homogenate supernatants were quantified using commercial enzyme-linked immunosorbent assay (ELISA) kits (TNF-α: Cat. No. CRE0004, IL-1β: Cat. No. CME0015, IL-6: Cat. No. CME0006; Beijing Sizhengbai Biotechnology Co., Ltd., China) according to the manufacturer’s instructions. To evaluate oxidative stress levels, the activities of superoxide dismutase (SOD, Cat. No. BC5165; Jiangsu MeiMian Industrial Co., Ltd., China) and glutathione peroxidase (GSH-Px, Cat. No. BC1195; Beijing Solarbio Science & Technology Co., Ltd., China), as well as the content of malondialdehyde (MDA, Cat. No. BC0025; Beijing Solarbio Science & Technology Co., Ltd., China), were measured using specific commercial assay kits in strict adherence to the provided protocols.

### Real-time quantitative PCR (RT-qPCR) analysis

Total RNA was extracted from colon tissues using the NucleoSpin^®^ RNA Plus (Cat. No. 9767, Takara Bio Inc., Shiga, Japan). Subsequently, RNA was reverse-transcribed into cDNA using the PrimeScript RT Reagent Kit (Cat. No. RR037A, Takara Bio Inc., Shiga, Japan). Using the synthesized cDNA as a template and β-actin as an internal reference gene, RT-qPCR was performed on a real-time PCR detection system with the SYBR^®^ Premix Ex Taq™ II (Cat. No. RR420A, Takara Bio Inc., Shiga, Japan). The primer sequences are listed in Supplementary Table 2.

### H&E staining and immunohistochemistry

Hematoxylin and eosin staining: a 2-cm segment of the distal colon was fixed in 4% paraformaldehyde, embedded in paraffin, and sectioned into 4-μm thick slices. The sections were deparaffinized in xylene and rehydrated through a graded ethanol series. Following this, nuclei were stained with hematoxylin for 5–10 min, differentiated in acid ethanol, and then cytoplasm was counterstained with eosin for 1–3 min. Finally, the sections were dehydrated through a graded ethanol series, cleared in xylene, and mounted with neutral gum.

Immunohistochemistry (IHC): after deparaffinization and rehydration, antigen retrieval was performed by heating the sections in citrate buffer (pH 6.0). Endogenous peroxidase activity was blocked by incubation with 3% hydrogen peroxide (H_2_O_2_), and non-specific binding sites were blocked with 5% bovine serum albumin (BSA). The sections were then incubated with primary antibodies against ZO-1 (dilution 1:50, Cat. No. HA722797), Occludin (dilution 1:100, Cat. No. R1510-33), Claudin-1 (dilution 1:150, Cat. No. RT1141), SOD1 (dilution 1:1500, Cat. No. ET1702-36), or SOD2 (dilution 1:2000, Cat. No. ET1701-54) at 4 °C overnight (All antibodies were purchased from Hangzhou HuaAn Biotechnology Co., Ltd., China). Subsequently, the sections were incubated with appropriate horseradish peroxidase (HRP)-conjugated secondary antibodies at room temperature for 30 min. Signal visualization was achieved using a 3,3′-diaminobenzidine (DAB) substrate, and nuclei were counterstained with hematoxylin. Finally, the sections were dehydrated, cleared, and mounted. Stained images were observed and captured using an inverted microscope (He et al., 2021; Huang et al., 2022). Immunohistochemical staining was quantified by measuring the integrated optical density (IOD) using ImageJ software, and the results were expressed as the mean optical density value per field.

### Short-chain fatty acid (SCFA) analysis

Approximately 0.1 g of intestinal content from each mouse was accurately weighed and homogenized with 1 mL of phosphate-buffered saline (PBS) to prepare a 10% (w/v) fecal suspension. Then, 500 μL of the suspension was mixed with 100 μL of crotonic acid in metaphosphoric acid solution, vortexed thoroughly, and stored at −20 °C for 24 h. After thawing at 4 °C, the samples were centrifuged. The resulting supernatant was filtered through a 0.45 μm hydrophilic filter membrane. Using crotonic acid as an internal standard, the short-chain fatty acid (SCFA) content in the samples was analyzed by gas chromatography (GC) system equipped with a DB-FFAP column (0.32 mm × 30 m × 0.5 μm, Agilent).

### 16 S rRNA high-throughput sequencing analysis

Total genomic DNA was extracted from the fecal samples. The full-length 16S rRNA gene was subsequently amplified by polymerase chain reaction (PCR) using barcoded universal primers (27F: 5′-AGRGTTTGATYNTGGCTCAG-3′ and 1492R: 5′-TASGGHTACCTTGTTASGACTT-3′) with the extracted DNA as template. The PCR amplicons were then purified, quantified, and normalized for SMRT Bell library construction. Sequencing was conducted on the PacBio Sequel II platform (Beijing Biomarker Technologies Co., Ltd.) according to the manufacturer’s standard protocol. Raw subreads were processed using SMRT Link (v8.0) to generate circular consensus sequencing (CCS) reads with the parameters minPasses ≥ 5 and minPredictedAccuracy ≥ 0.9. CCS reads were demultiplexed by lima (v1.7.0) according to barcode sequences. Primers were identified and removed using cutadapt (v2.7) with a maximum error rate of 0.2, reads lacking both forward and reverse primers were discarded. Length filtering was then applied to retain CCS sequences between 1200 and 1650 bp. Chimeric sequences were identified and removed using UCHIME (version 8.1). Each query sequence is split into non-overlapping chunks, and the best hits for each chunk in a reference database are used to identify the two most likely parent sequences. The query is then considered chimeric if a fragment sharing >80% similarity with the query is found on both parents. A total of 1429763 CCS reads were generated from the 24 samples following sequencing and barcode demultiplexing, where the minimum CCS read count per sample was 42811 and the mean read count reached 59573. The DADA2 pipeline in QIIME2 (v2020.6) was applied to denoise the reads, and ASVs below the 0.005% of the total sequencing reads threshold were excluded. A total of 1306 ASVs were detected across the 24 samples, with per-sample richness ranging from 167 to 542 ASVs. Taxonomic annotation was using the blast-based consensus method with the parameter set as minimum similarity in sequence 90%; minimum coverage 90%, minimum consensus 51%. The ones that could not be matched in the reference database are further classified by Naive Bayes classifier against reference databases such as Silva or Greengenes, with parameters including ≥90% similarity, ≥90% coverage, and a confidence threshold of 0.7. The diversity, differential analysis between groups was analyzed on the Biomarker Cloud Platform^1^. The alpha-diversity indices, including Shannon and Simpson were calculated to estimate microbiota diversity and abundance in each sample using QIIME2 (v2020.6) software. Beta diversity was assessed based on the Bray-Curtis dissimilarity index and visualized via principal coordinates analysis (PCoA).

### Untargeted metabolomics analysis

Fecal specimens (50 mg) were homogenized with a methanol-acetonitrile-water (2:2:1, v/v/v) extraction solvent using an ultrasonic-assisted extraction method, followed by protein precipitation and centrifugal separation. Metabolite profiling was performed using an ultra-high performance liquid chromatography-mass spectrometry (UHPLC-MS) system at Biomarker Technologies Co., Ltd., (Beijing, China). The UHPLC-MS system consisted of a Waters Acquity I-Class PLUS ultra-high performance liquid chromatograph coupled with a Waters Xevo G2-XS QTof high-resolution mass spectrometer. Separation was achieved using an Acquity UPLC HSS T3 column (100 mm × 2.1 mm, 1.8 μm). The Waters Xevo G2-XS QTof high-resolution mass spectrometer can collect primary and secondary mass spectrometry data in MS^E^ mode under the control of the acquisition software (MassLynx V4.2, Waters). In each data acquisition cycle, dual-channel data acquisition can be performed simultaneously at low collision energy and high collision energy. The low collision energy was set at 6 eV, while the high collision energy ranged from 10 to 40 eV, with a scan rate of 0.2 s per spectrum. The parameters for the ESI ion source were as follows: capillary voltage: 2500 V (positive ion mode) or −2000 V (negative ion mode); cone voltage: 30 V; ion source temperature: 100 °C; solvent gas temperature: 500 °C; cone gas flow rate: 50 L/h; desolvation gas flow rate: 800 L/h. Raw data acquisition and processing were conducted on the Biomarker Cloud Platform^2^. The raw data collected using MassLynx V4.2 were processed using Progenesis QI software for peak extraction, peak alignment, and other data processing operations. Identification is performed using the Progenesis QI software with the online METLIN database, public databases, and a self-built database. Theoretical fragment identification is also conducted.

### Data processing and statistical analysis

Statistical analysis and graphs were generated using GraphPad Prism 10.1.2. Prior to inter-group comparisons, the normality of data distribution was assessed using the Shapiro–Wilk test, and homogeneity of variances was evaluated using the Brown–Forsythe test. Since the data met the assumptions of normality and homogeneity of variance, inter-group comparisons were conducted using one-way ANOVA followed by Tukey’s multiple comparisons test. A *P*-value of less than 0.05 was considered statistically significant.

## Results

### Effects of TLYY administration on body weight and pathological features in DSS-induced mice

**FIGURE 1:**
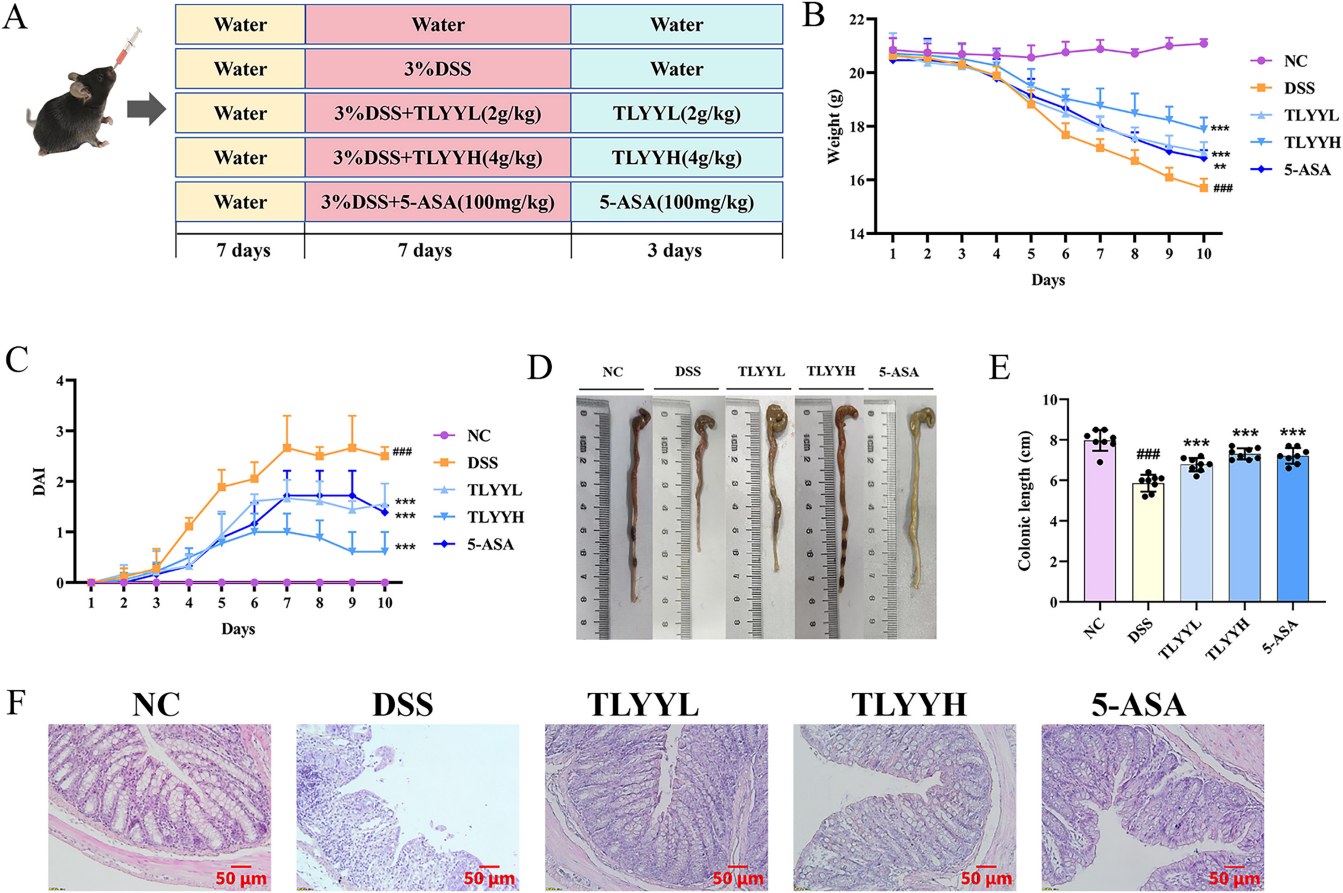
Taoling Yuyang Decoction (TLYY) improved the clinical symptoms of UC mice induced by DSS. **(A)** Experimental design; **(B)** changes in body weight of mice; **(C)** DAI index; **(D,E)** the length of the colon in mice; **(F)** representative histopathological changes in the colon tissues of mice (H&E stain with a scale bar of 50 μm). Data are expressed as means ± SD (*n* = 8), with compared to NC, ### represents *p* < 0.001; compared to DSS * represents *p* < 0.05, ** represents *p* < 0.01, *** represents *p* < 0.001, and ns represents *p* > 0.05.

The DSS-induced colitis mouse model closely recapitulates the symptomatic manifestations of human ulcerative colitis, including reduced activity, body weight loss, diarrhea, and fecal occult blood. The experimental timeline is illustrated in Figure 1A. Results showed that mice began to develop clinical signs of UC 3 days after DSS administration, as evidenced by progressive weight loss, stool softening, and fecal occult blood, which collectively resulted in a steadily increasing disease activity index (DAI) score (Figures 1B,C). These collective findings confirm the successful induction of colitis in the experimental model. From day 5 onward, all DSS-treated groups exhibited significant weight loss compared with the NC group, with the most severe reduction observed in the DSS model group. Treatment with a high dose of TLYY (TLYYH) significantly alleviated DSS-induced weight loss, and this protective effect was superior to that of the low-dose TLYY (TLYYL) and 5-ASA groups. Colon length shortening is a reliable indicator of intestinal inflammation and tissue damage. The measurements revealed a significant reduction in colon length in the DSS model group. In contrast, both TLYY-treated groups markedly ameliorated this DSS-induced colon shortening, with the TLYYH group showing a more pronounced effect (Figures 1D, E). Histopathological analysis of colon tissue by H&E staining (Figure 1F) demonstrated an intact colonic architecture in the NC group, characterized by well-structured crypts, abundant goblet cells, and the absence of inflammatory cell infiltration. Conversely, the DSS group exhibited severe mucosal damage, including crypt destruction, depletion of goblet cells, and extensive inflammatory cell infiltration. Treatment with TLYY, particularly at the high dose, substantially restored crypt architecture and intestinal integrity, increased goblet cell numbers, and reduced inflammatory cell infiltration. These findings indicate that TLYY confers a dose-dependent protective effect against DSS-induced colonic injury.

### Effects of TLYY administration on oxidative stress levels in DSS-induced mice

**FIGURE 2:**
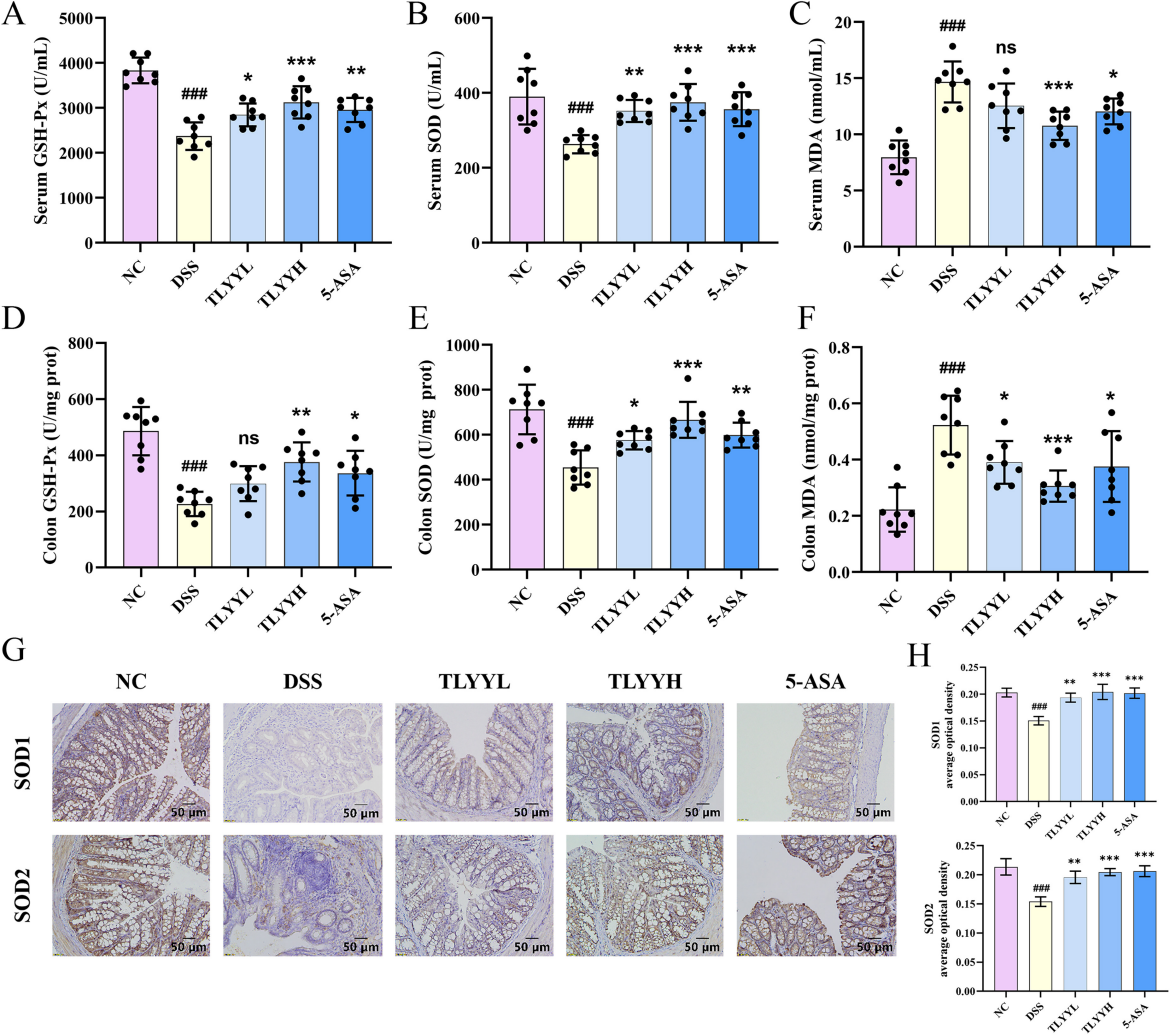
Taoling Yuyang Decoction (TLYY) reduced the oxidative stress level in mice induced by DSS. **(A–C)** The levels of GSH-Px, SOD and MDA in the serum of mice; **(D–F)** the levels of GSH-Px, SOD and MDA in the colon tissues; **(G)** immunohistochemical results of SOD1 and SOD2 in the colon tissue of mice with a scale bar of 50 μm; **(H)** quantification of SOD1 and SOD2. Data are expressed as means ± SD (*n* = 8), with compared to NC, ### represents *p* < 0.001; compared to DSS * represents *p* < 0.05, ** represents *p* < 0.01, *** represents *p* < 0.001, and ns represents *p* > 0.05.

As shown in Figures 2A–C, DSS induction significantly decreased serum GSH-Px and SOD activities and increased MDA content compared to the NC group. Treatment with both TLYYL and TLYYH significantly restored GSH-Px and SOD activities, with the TLYYH group also demonstrating a significant reduction in MDA levels. Consistent with these findings, analysis of colonic oxidative stress markers (Figures 2D–F) revealed that DSS administration led to significantly reduced SOD and GSH-Px activities and elevated MDA content relative to the NC group. Compared to the DSS group, the TLYYH group significantly enhanced GSH-Px activity, while TLYYL and TLYYH groups significantly increased SOD activity and decreased MDA content. To further investigate the effect of TLYY on SOD isoforms, SOD1 and SOD2 protein expression in colon tissue was examined by immunohistochemistry. The results showed significantly decreased expression of both SOD isoforms in the DSS group compared to the NC group, while both TLYY doses markedly upregulated their expression levels (Figures 2G,H). Collectively, these results demonstrate that high-dose TLYY administration effectively mitigates systemic oxidative stress and exhibits potent antioxidant activity.

### Effects of TLYY administration on inflammatory cytokine levels in DSS-induced mice

**FIGURE 3:**
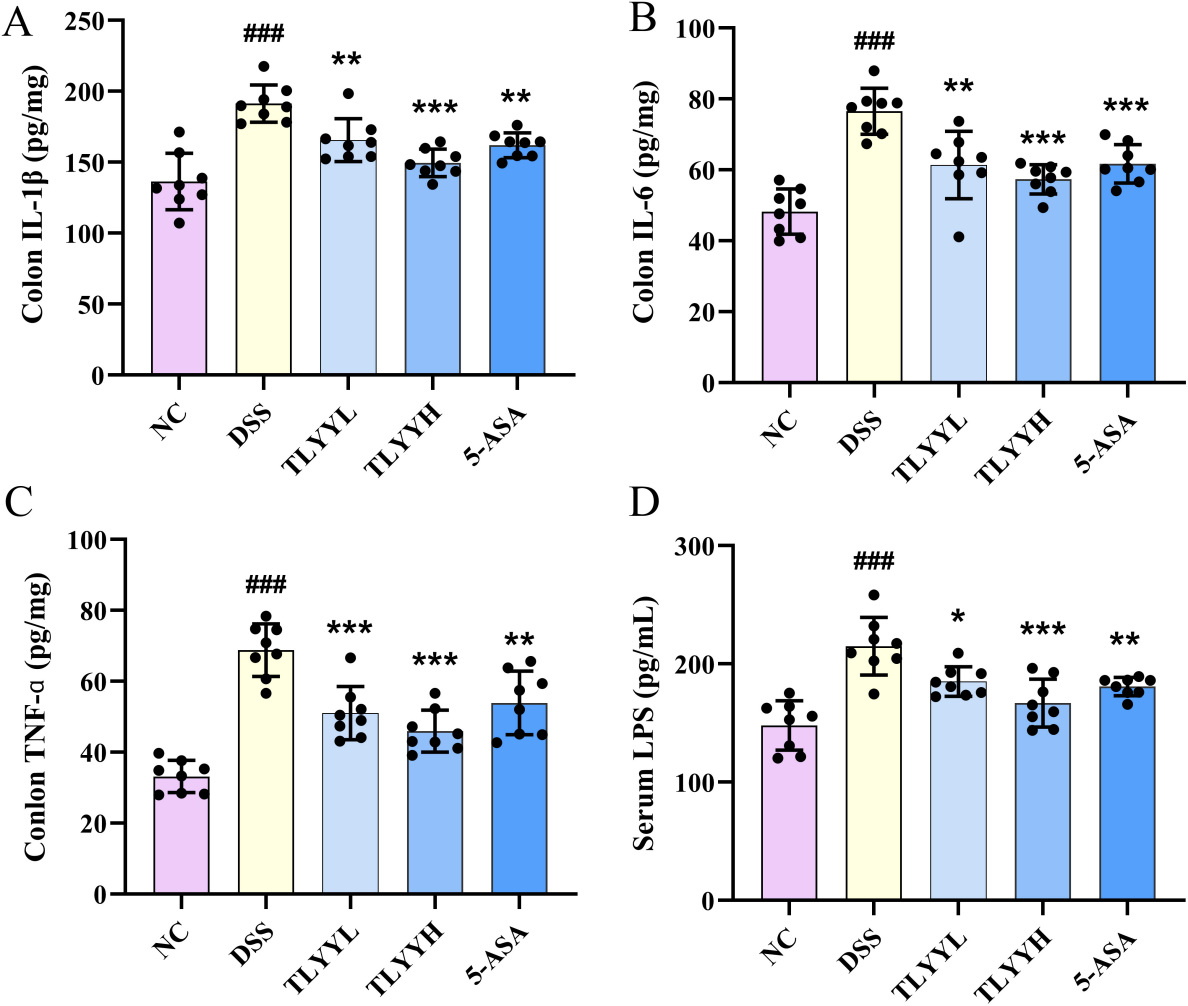
Taoling Yuyang Decoction (TLYY) reduced the levels of inflammatory factors in mice induced by DSS. **(A–C)** The levels of IL-1β, IL-6, and TNF-α in the colon tissues; **(D)** the level of LPS in the serum of mice. Data are expressed as means ± SD (*n* = 8), with compared to NC, ### represents *p* < 0.001; compared to DSS * represents *p* < 0.05, ** represents *p* < 0.01, *** represents *p* < 0.001, and ns represents *p* > 0.05.

The therapeutic effect of TLYY on colitis was evaluated by measuring inflammatory cytokine levels in mouse colon tissue. As shown in Figures 3A–C, DSS induction significantly increased the levels of TNF-α, IL-6, and IL-1β compared to the NC group. Both TLYYL and TLYYH treatments significantly attenuated this DSS-induced upregulation of pro-inflammatory cytokines. TLYYH showed superior anti-inflammatory effects to TLYYL and comparable to the 5-ASA group. Given that DSS-induced ulcerative colitis disrupts the intestinal barrier, permitting the translocation of intestinal endotoxin into systemic circulation, we measured serum LPS levels (Figure 3D). DSS administration resulted in a significant increase in serum LPS compared to the NC group. TLYY two-dose treatment regimen significantly reduced serum LPS levels, with the TLYYH group demonstrating the most pronounced effect. These findings collectively indicate that TLYY administration effectively mitigates DSS-induced intestinal inflammation and reduces systemic endotoxemia in mice.

### Protective effects of TLYY administration on intestinal permeability in DSS-induced mice

**FIGURE 4:**
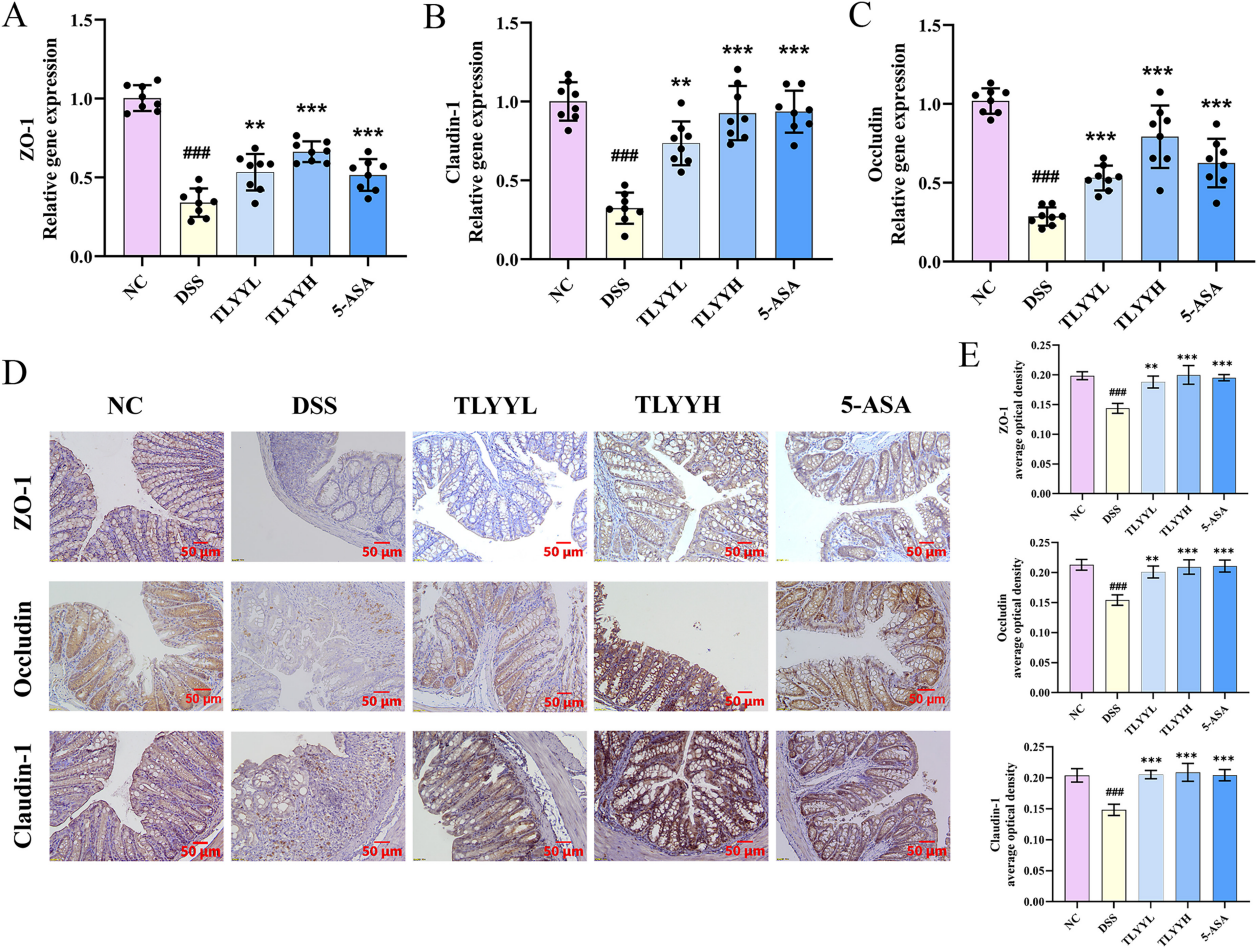
Impact of TLYY on intestinal permeability proteins in DSS-induced mice. The expression of Zonula Occludens-1 (ZO-1) **(A)**, Claudin-1 **(B)** and Occludin **(C)** in colon tissues was detected by RT-qPCR; **(D)** immunohistochemical results of ZO-1, Claudin-1, and Occludin in the colon tissues with a scale bar of 50 μm and **(E)** the quantification of ZO-1, Claudin-1, and Occludin. Data are expressed as means ± SD (*n* = 8), with compared to NC, ### represents *p* < 0.001; compared to DSS ** represents *p* < 0.01, *** represents *p* < 0.001, and ns represents *p* > 0.05.

A hallmark pathological alteration in ulcerative colitis is the disruption of intestinal barrier integrity. Analysis of genes associated with intestinal permeability revealed that DSS induction significantly downregulated the expression of ZO-1, Occludin, and Claudin-1 compared to the NC group (Figures 4A–C). Both low-dose and high-dose TLYY treatments significantly restored the gene expression levels of these tight junction proteins. Consistent with the mRNA expression result, immunohistochemical analysis demonstrated a substantial reduction in ZO-1, Occludin, and Claudin-1 protein expression in the DSS group, which was effectively reversed by TLYY treatment (Figures 4D,E). The therapeutic effect on barrier protein restoration was more pronounced in the TLYYH group. These collective results demonstrate that TLYY administration promotes the repair of the intestinal barrier and ameliorates intestinal damage in DSS-induced colitis.

### Effect of TLYY administration on gut microbiota in DSS-induced mice

**FIGURE 5:**
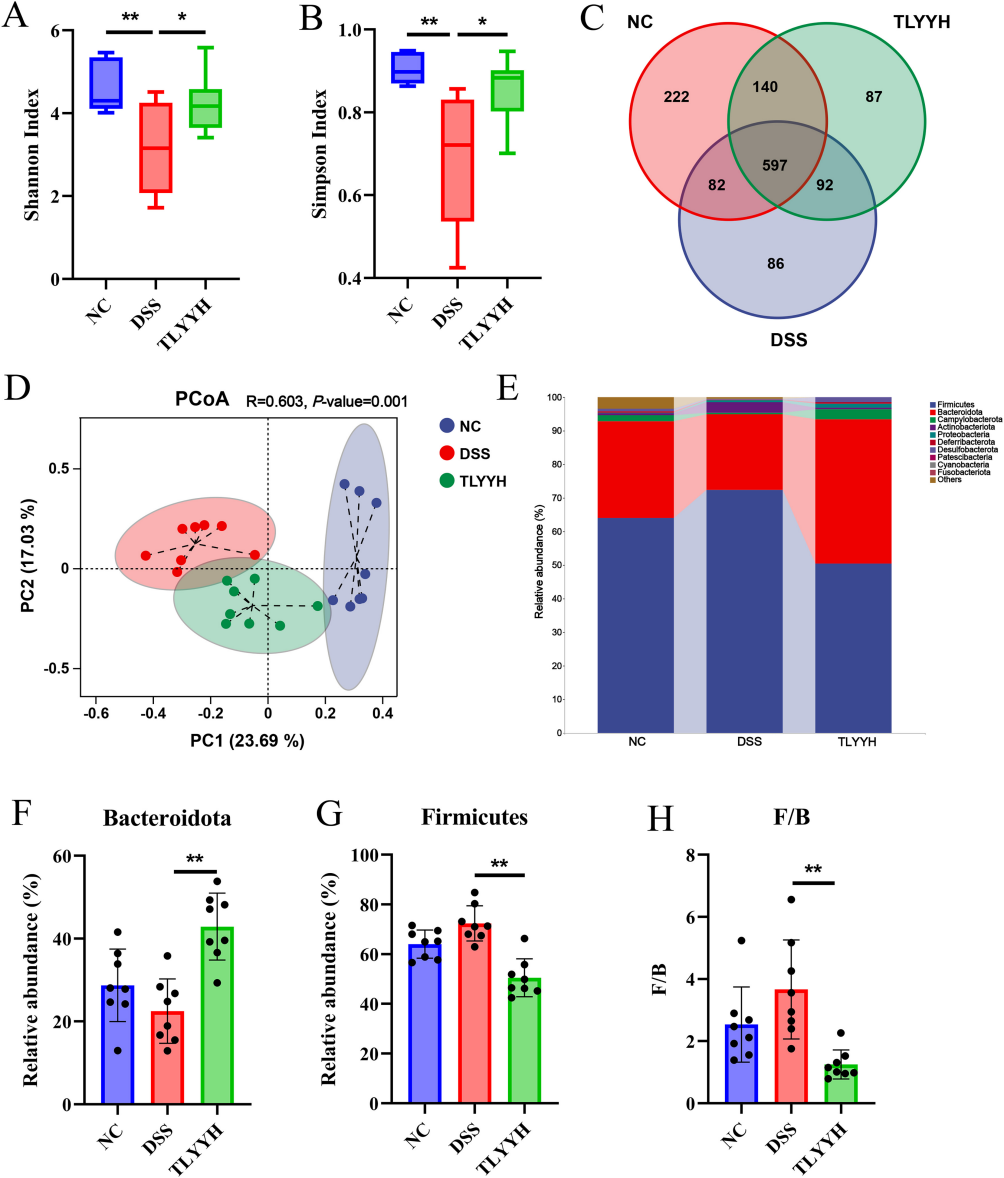
The impact of TLYY on the diversity and composition of the intestinal microbiota in DSS - induced mice. **(A,B)** Alpha diversity analysis of Shannon and Simpson index; **(C)** venn diagrams of the three groups of species; **(D)** beta diversity analysis using PCoA based on Bray - Curtis distance; **(E)** bacterial taxonomic profiling in the phylum level, **(F)** the relative abundance of Bacteroidota; **(G)** the relative abundance of Firmicutes; **(H)** ratio of Firmicutes/Bacteroidetes (F/B). Data are expressed as means ± SD (*n* = 8), with* represents *p* < 0.05, ** represents *p* < 0.01.

Natural active products can ameliorate ulcerative colitis (UC) through modulation of the gut microbiota. Based on the above results, which demonstrate that the therapeutic effect of the high-dose treatment group is superior to that of the low-dose group, we conducted gut microbiota analysis on the NC, DSS, and TLYYH groups. After performing full-length 16S rDNA sequencing analysis on the 24 samples and identifying sequences by barcode, a total of 1,429,752 Circular Consensus Sequencing (CCS) reads were obtained. Each sample generated at least 42,811 CCS reads, with an average of 59,573 reads per sample. Alpha diversity analysis (Shannon and Simpson index) demonstrated that TLYY significantly restored the DSS-induced reduction in microbial diversity (Figures 5A,B). Venn diagram analysis (Figure 5C) illustrated the distribution of operational taxonomic units (OTUs) among groups, with the NC, DSS, and TLYYH groups containing 222, 86, and 87 unique OTUs, respectively, while sharing 597 core OTUs, indicating structural reorganization of the gut microbiota following TLYYH intervention. Principal coordinates analysis (PCoA) based on Bray-Curtis distance (Figure 5D) revealed distinct clustering patterns among the three groups, with the TLYYH group showing closer proximity to the NC group, further confirming the structural modulation by TLYYH. Microbial composition analysis (Figure 5E) identified Firmicutes, Bacteroidota, Campylobacterota, and Actinobacteriota as the dominant phyla, collectively representing >95% of the total abundance. Compared to the DSS group (Figures 5F–H), the TLYYH group showed a significant decrease in Firmicutes abundance and a marked increase in Bacteroidota. Consequently, the Firmicutes/Bacteroidota (F/B) ratio, which was elevated in the DSS group, was significantly reduced by TLYYH treatment. These results indicate that TLYYH effectively counteracts DSS-induced dysbiosis by restoring the balance between dominant bacterial phyla and promoting microbial homeostasis.

**FIGURE 6:**
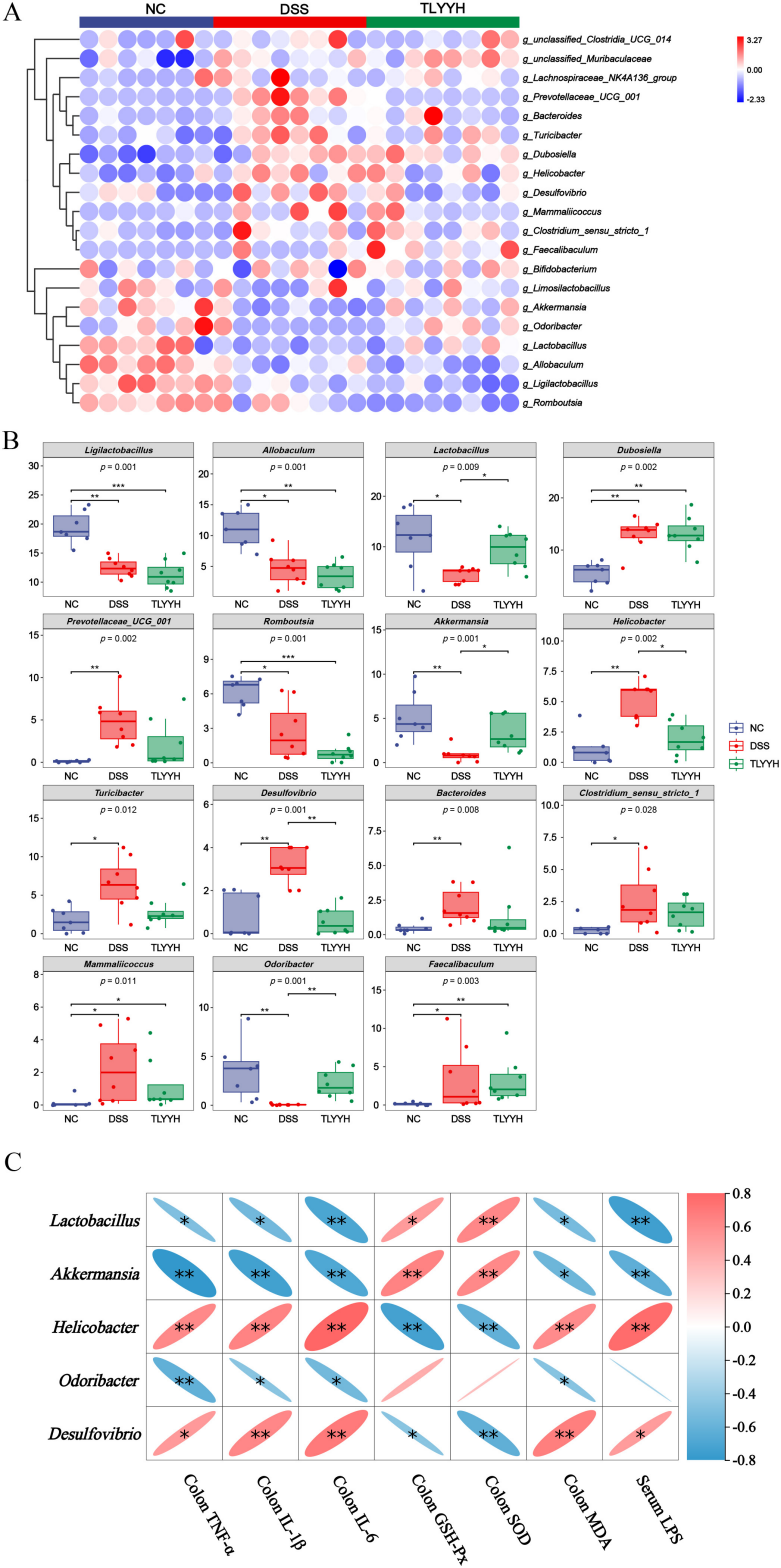
The impact of TLYYH on the intestinal microbiota of mice induced by DSS. **(A)** Hierarchical species abundance clustering heatmap at genue level; **(B)** analysis of differential bacteria species screening; **(C)** the correlation between the intestinal microbiota and the inflammatory factors (IL-1β, IL-6, and TNF-α in the colon tissues), oxidative stress indicators (GSH-Px, SOD and MDA in the colon tissues) and LPS in serum induced by DSS. Positive correlations are depicted in red, whereas negative correlations are shown in blue. Data are expressed as means ± SD (*n* = 8), with* represents *p* < 0.05, ** represents *p* < 0.01, *** represents *p* < 0.001.

Further analysis of the impact of TLYYH on the gut microbiota in DSS-induced mice revealed distinct differences in bacterial distribution at the genus level. The species abundance heatmap (Figure 6A) illustrates these intergroup variations. The microbial abundance pattern in the DSS group was markedly different from that in the NC group. Following TLYYH intervention, the abundances of several bacterial genera shifted toward the NC group pattern, suggesting that TLYYH can modulate the gut microbiota structure. Analysis of differentially abundant genera (Figure 6B) showed that, compared to the NC group, the DSS group exhibited significant alterations in the relative abundances of multiple genera, such as *Ligilactobacillus*, *Allobaculum*, *Lactobacillus*, *Romboutsia*, *Akkermansia*, and *Odoribacter* were significantly decreased. Conversely, the relative abundances of genera including *Dubosiella*, *Prevotellaceae_UCG_001*, *Helicobacter*, *Turicibacter*, *Desulfovibrio*, *Bacteroides*, *Clostridium*_*sensu*_*stricto*_*1*, *Mammaliicoccus*, and *Faecalibaculum* were significantly increased. TLYYH intervention significantly reversed these changes, increasing the relative abundances of *Lactobacillus*, *Akkermansia*, and *Odoribacter*, while significantly reducing the abundances of *Helicobacter* and *Desulfovibrio*.

A correlation analysis was conducted to investigate the relationships between gut microbiota and indicators of inflammatory injury and oxidative stress induced by DSS. The findings (Figure 6C) suggest that *Lactobacillus* and *Akkermansia*, which are considered potentially beneficial genera, exhibited a significant negative correlation with colonic pro-inflammatory cytokines (TNF-α, IL-1β, IL-6), MDA and serum LPS, and a significant positive correlation with colonic antioxidant indicators (GSH-Px, SOD). Likewise, *Odoribacter* also demonstrated significant negative correlations with colonic TNF-α, IL-1β, IL-6, and MDA levels. In contrast, potentially harmful genera such as *Helicobacter* and *Desulfovibrio* showed significant positive correlations with the level of TNF-α, IL-1β, IL-6, MDA and LPS, and significant negative correlations with antioxidant indicators (GSH-Px, SOD), indicating that these genera may exacerbate intestinal inflammation and oxidative damage.

### Effects of TLYY administration on short-chain fatty acids in DSS-induced mice

**FIGURE 7:**
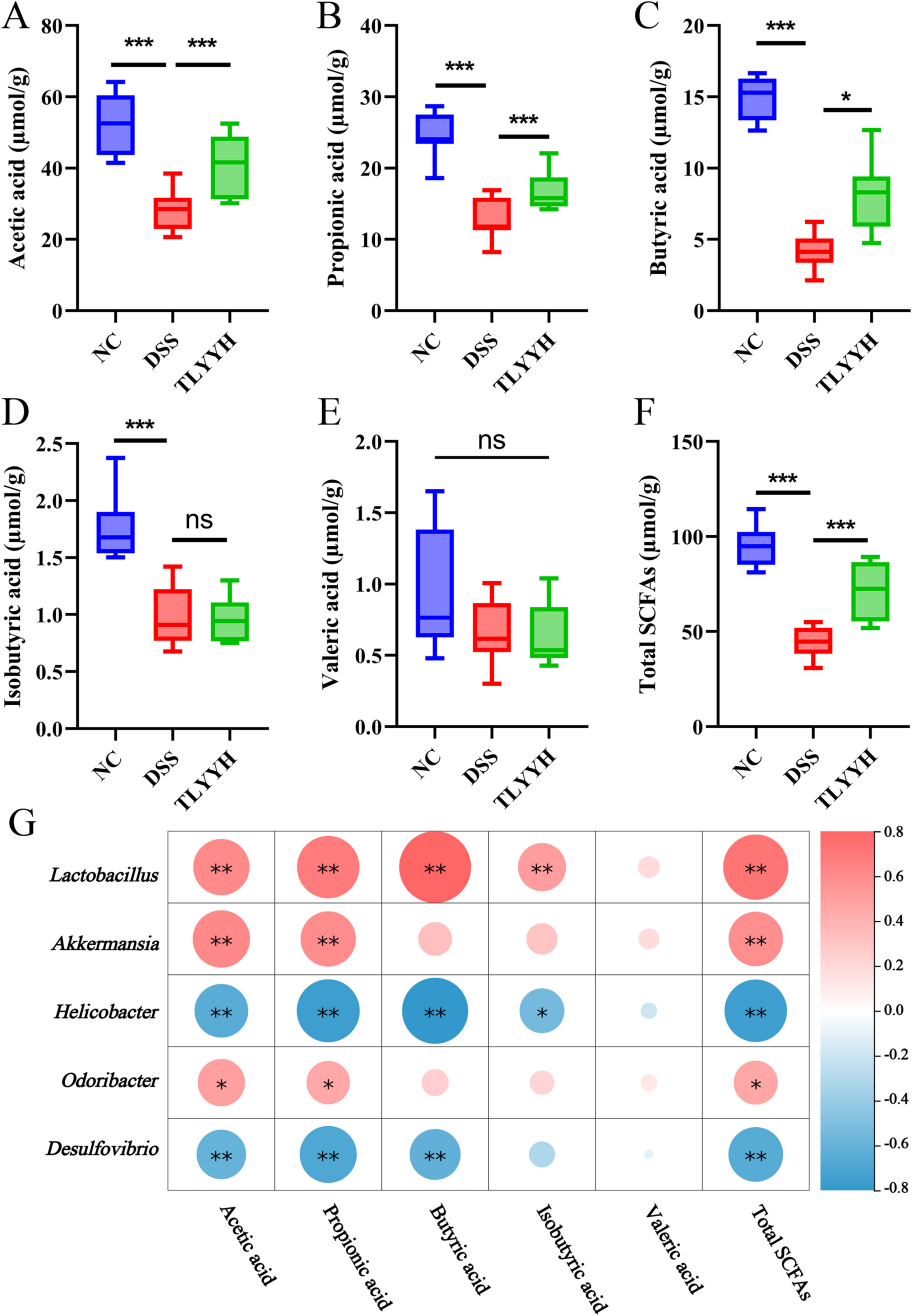
The effect of TLYY on short-chain fatty acids induced by DSS in mice. **(A)** acetic acid; **(B)** propionic acid; **(C)** butyric acid; **(D)** isobutyric acid; **(E)** valeric acid; **(F)** total SCFAs; **(G)** Spearman correlation analysis. Positive correlations are depicted in red, whereas negative correlations are shown in blue. Data are expressed as means ± SD (*n* = 8), with* represents *p* < 0.05, ** represents *p* < 0.01, *** represents *p* < 0.001, and ns represents *p* > 0.05.

Research has established that short-chain fatty acids (SCFAs) modulate mucosal immunity and intestinal homeostasis through receptors expressed on immune and epithelial cells, with the colon showing prominent expression in both intestinal epithelial cells and inflammatory cells (Tan et al., 2023). A distinctive feature of UC and other colonic disorders is a significant decrease in intestinal short-chain fatty acid (SCFA) levels. To assess the impact of TLYY on intestinal homeostasis and inflammation in DSS-induced colitis, the SCFA levels in intestinal contents were quantified. As shown in Figures 7A–F, compared to the NC group, the DSS group exhibited significantly decreased levels of acetic acid, propionic acid, butyric acid, isobutyric acid, and total SCFAs. In contrast, TLYYH treatment significantly restored the concentrations of acetic acid, propionic acid, butyric acid, and total SCFAs.

Spearman correlation analysis was conducted to assess the relationship between gut microbiota composition and SCFA levels across experimental groups. The results (Figure 7G) indicated that the relative abundances of *Lactobacillus*, *Akkermansia*, and *Odoribacter* were significantly positively correlated with the contents of acetic acid, propionic acid, and total SCFAs. Additionally, *Lactobacillus* abundance showed a significant positive correlation with butyric acid and isobutyric acid levels. Conversely, the relative abundances of *Helicobacter* and *Desulfovibrio* were significantly negatively correlated with acetic acid, propionic acid, butyric acid, and total SCFAs. Integrating these findings with the previously observed microbial shifts, we propose that TLYY intervention alleviates murine colitis by modulating gut microbiota composition, specifically by promoting beneficial bacteria and suppressing harmful bacteria, which in turn enhances SCFA production and metabolic homeostasis.

### Effects of TLYY administration on fecal metabolites in DSS-induced mice

To comprehensively characterize metabolic alterations in response to different interventions, we conducted untargeted metabolomic profiling of fecal samples. This project was based on the LC-QTOF platform and involved metabolomics qualitative and quantitative analysis of 24 samples. This report presents the metabolites detected under the default mode, resulting in the detection of 8,588 peaks, with annotation of 2,519 metabolites. Principal coordinates analysis (PCoA) was performed to visualize the global metabolic profiles under both positive and negative ion modes (Supplementary Figure 1). The results demonstrated clear separation among the three groups, with the TLYYH group exhibiting a spatial distribution pattern closer to the NC group than to the DSS group in both ionization modes. However, in the negative ion mode, although the TLYYH cluster shifted toward the NC group, it remained partially overlapping with the DSS group, indicating distinct yet incompletely resolved metabolic differences among the three groups.

**FIGURE 8:**
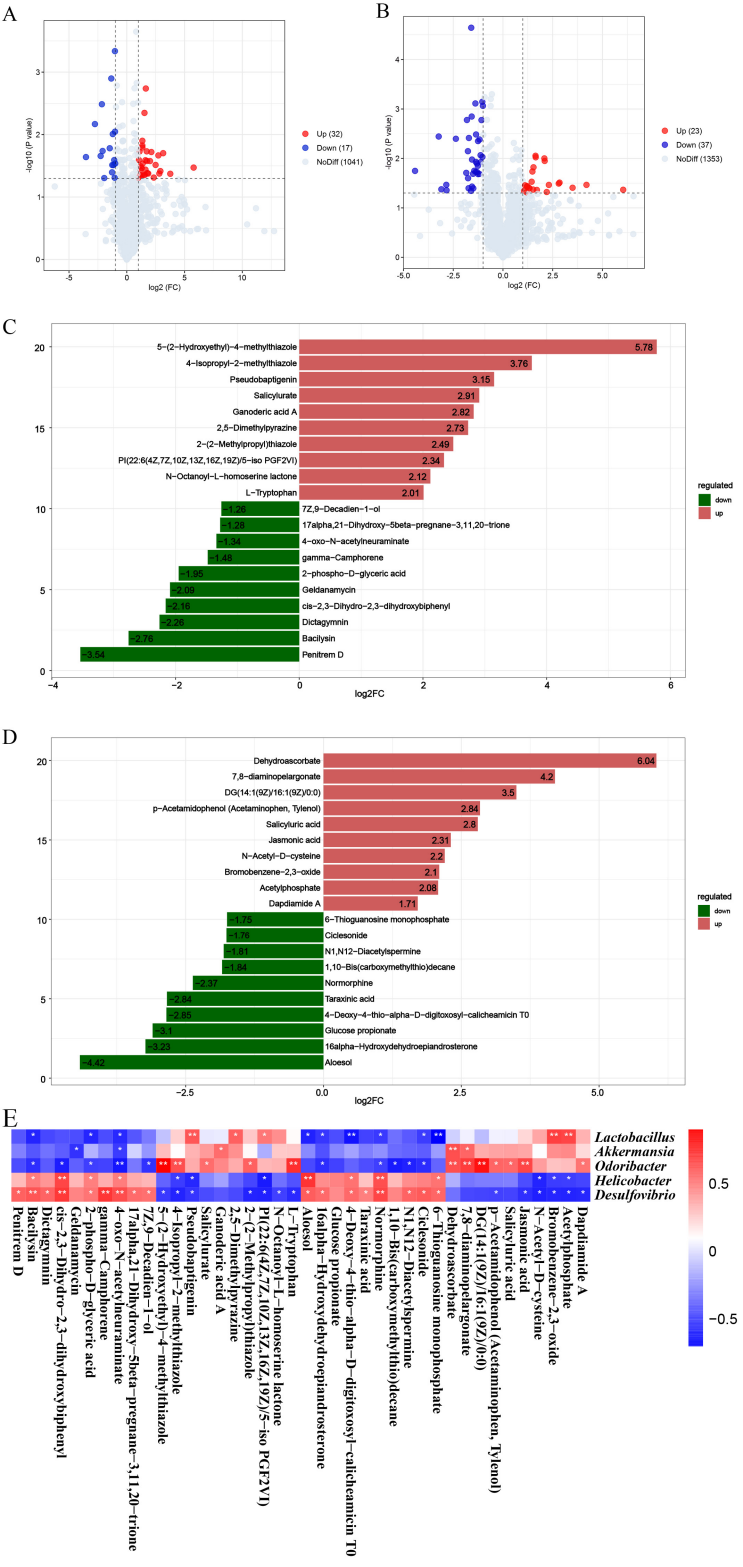
The fecal metabolic profile altered by TLYYH in DSS mice. Differential metabolites in fecal identification between DSS and TLYY in panels **(A,C)** positive and **(B,D)** negative ion modes. **(A,B)** Volcano plot, **(C,D)** fold-change bar chart. **(E)** Correlation heatmap of differential microbiota and serum metabolites. Data were calculated by Spearman’s correlation method after mean centering and unit variance scaling. *p < 0.05, **p < 0.01.

Volcano plots were used to visualize the overall trends of metabolite differences between two groups and the statistical significance of these differences. Univariate analysis incorporating fold change, *t*-test, and variable importance in projection (VIP) filtering revealed that, compared to the NC group, the DSS group exhibited significant changes in 57 metabolites in positive ion mode (26 upregulated, 31 downregulated; Supplementary Figure 2) and 68 metabolites in negative ion mode (36 upregulated, 32 downregulated; Supplementary Figure 2). Compared to the DSS group, the TLYYH group showed significant alterations in 49 metabolites in positive ion mode (32 upregulated, 17 downregulated) and 60 metabolites in negative ion mode (23 upregulated, 37 downregulated; Figures 8A,B).

This study analyzed the top 10 significantly upregulated and downregulated metabolites in each group. The fold-change bar chart comparing the NC and DSS groups (Supplementary Figure 2) showed that in positive ion mode, the metabolite with the highest increase in the DSS-induced model group was 1alpha,3beta,22R-Trihydroxyergosta-5,24E-dien-26-oic acid 3-O-b-D-glucoside26-O-[b-D-glucosyl-(1- > 4)-b-D-glucosyl-(1- > 2)-6-acetyl-b-D-glucosyl] ester (log2FC = 36.37), while the metabolite with the highest decrease was Psychosine (log2FC = −6.55). In negative ion mode (Supplementary Figure 2), the metabolite with the highest increase in the DSS group was Glucoputranjivin (log2FC = 3.77), and the metabolite with the highest decrease was Piperonal (log2FC = −4.56). Following TLYYH treatment, in positive ion mode (Figure 8C), metabolites with relatively high fold increases included 5-(2-Hydroxyethyl)-4-methylthiazole, 4-Isopropyl-2-methylthiazole, Pseudobaptigenin, Salicylurate, and Ganoderic acid A. Metabolites with relatively high fold decreases included Penitrem D, Bacilysin, and Dictagymnin. In negative ion mode (Figure 8D), metabolites with relatively high fold increases included Dehydroascorbate, 7,8-diaminopelargonate and DG(14:1(9Z)/16:1(9Z)/0:0), while those with relatively high fold decreases included Aloesol, 16alpha-Hydroxydehydroepiandrosterone and Glucose propionate. Notably, in positive ion mode, TLYYH significantly downregulated the DSS-induced elevation of metabolites such as Geldanamycin and Bacilysin (Figure 8C, Supplementary Figure 2), suggesting these metabolites may play important roles in the mechanism by which TLYY ameliorates UC.

The altered fecal metabolic profile may reflect the functions of the gut microbiota. Here, Spearman’s correlation analysis was employed to explore the functional correlations between the significantly changed microbiota and the top 40 metabolites significantly enriched in KEGG pathways (Figure 8E). The results showed that *Lactobacillus* was significantly positively correlated with Pseudobaptigenin, 2,5-Dimethylpyrazine, Bromobenzene-2,3-oxide, and Acetylphosphate. Both *Lactobacillus* and *Odoribacter* were significantly negatively correlated with Bacilysin, 2-phospho-D-glyceric acid, 4-oxo-N-acetylneuraminate, 16α-Hydroxydehydroepiandrosterone, Normorphine, and Ciclesonide. Additionally, *Akkermansia* was positively correlated with Ganoderic acid A, Dehydroascorbate, and 7,8-diaminopelargonate, while being negatively correlated with Geldanamycin and 4-oxo-N-acetylneuraminate. Both *Helicobacter* and *Desulfovibrio* were significantly positively correlated with Bacilysin, cis-2,3-Dihydro-2,3-dihydroxybiphenyl, 2-phospho-D-glyceric acid, Aloesol, 4-Deoxy-4-thio-alpha-D-digitoxosyl-calicheamicin T0, Normorphine, and 6-Thioguanosine monophosphate, and negatively correlated mainly with 4-Isopropyl-2-methylthiazole, Pseudobaptigenin, N-Acetyl-D-cysteine, Bromobenzene-2,3-oxide, and Acetylphosphate.

**FIGURE 9:**
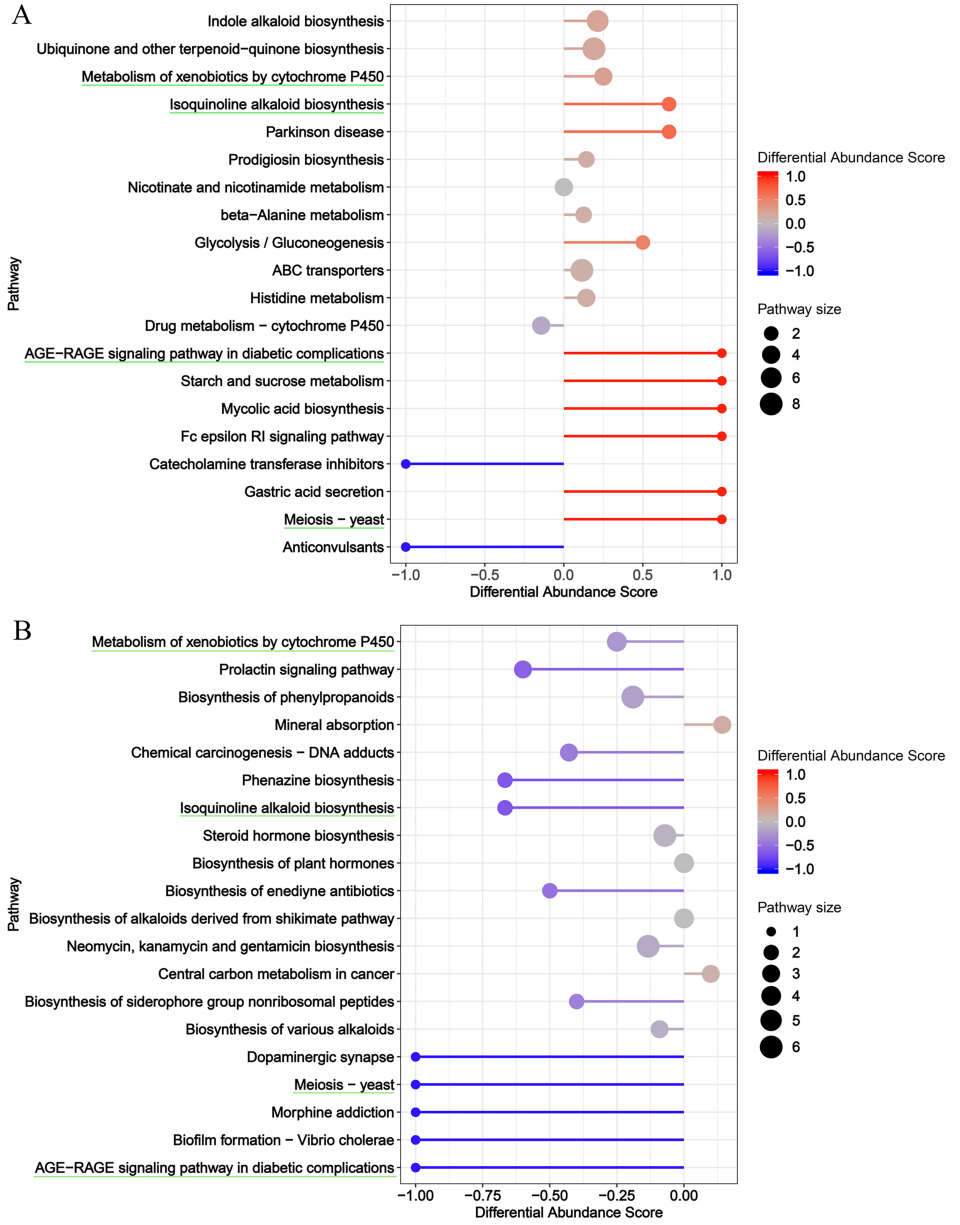
The enrichment metabolic pathway of fecal altered metabolites by Kyoto encyclopedia of genes and genomes (KEGG) analysis, **(A)** DSS compare to NC, **(B)** TLYY compare to DSS.

Kyoto encyclopedia of genes and genomes functional annotation and enrichment analysis of the differential metabolites identified 20 significantly enriched pathways between the control and model groups (Figure 9). The enrichment bubble plots demonstrated that DSS induction substantially altered the fecal metabolic profiles compared to the NC group (Figure 9A). The most prominently upregulated pathways included the AGE-RAGE signaling pathway in diabetic complications, Starch and sucrose metabolism, Mycolic acid biosynthesis, Fc epsilon RI signaling pathway, Gastric acid secretion and Meiosis-yeast. Several core metabolic pathways also showed a high proportion of upregulated metabolites, such as Indole alkaloid biosynthesis, Ubiquinone and other terpenoid-quinone biosynthesis, Metabolism of xenobiotics by cytochrome P450, ABC transporters and Histidine metabolism. In contrast, pathways including Catecholamine transferase inhibitors and Anticonvulsants were enriched with a higher number of downregulated metabolites. Compared to the DSS group (Figure 9B), the TLYY intervention group exhibited a greater number of upregulated metabolites annotated to Mineral absorption and Central carbon metabolism in cancer pathways. In contrast, the most markedly downregulated pathways included Dopaminergic synapse, Meiosis-yeast, Morphine addiction, Biofilm formation-*Vibrio cholerae* and the AGE-RAGE signaling pathway in diabetic complications. Several core metabolic pathways also showed a high proportion of downregulated metabolites, such as Metabolism of xenobiotics by cytochrome P450 and Neomycin, kanamycin and gentamicin biosynthesis. Notably, four metabolic pathways include Metabolism of xenobiotics by cytochrome P450, Isoquinoline alkaloid biosynthesis, AGE−RAGE signaling pathway in diabetic complications, and Meiosis – yeast were identified as overlapping across the pairwise comparisons, suggesting their potential key roles in the therapeutic regulation of UC by TLYY.

## Discussion

Ulcerative colitis (UC) is a chronic non-specific inflammatory bowel disease characterized by a complex pathogenesis involving multiple interrelated processes, including intestinal barrier disruption, oxidative stress imbalance, excessive inflammatory activation, gut microbiota dysbiosis, and metabolic disturbances (Sahu et al., 2025; Li et al., 2022). Taoling Yuyang Decoction (TLYY) is a traditional Chinese medicine formula with documented efficacy in treating gastrointestinal disorders. Our previous clinical study demonstrated that TLYY significantly alleviates clinical symptoms in UC patients, with superior therapeutic outcomes compared to mesalazine enteric-coated tablets (Li, 2023). Nevertheless, the specific mechanisms underlying its therapeutic effects are not fully understood. To bridge this knowledge gap, the present study systematically evaluated the interventional effects of TLYY and explored its mechanistic basis in a DSS-induced mice model of UC. The investigation focused specifically on three interconnected pathological aspects: gut microbiota composition, oxidative stress status, and fecal metabolic profile. These findings are expected to provide experimental evidence supporting the clinical application of TLYY for UC management.

Body weight loss, colon shortening, and histological damage are hallmark pathological features of the DSS-induced UC model and serve as key metrics for evaluating therapeutic interventions. In this study, DSS induction could lead to significant body weight reduction, elevated DAI scores, and severe colonic damage, including crypt destruction, goblet cell depletion, and inflammatory cell infiltration. In contrast, treatment with TLYY effectively reversed these pathological alterations. Notably, the high-dose TLYY group demonstrated more pronounced effects in suppressing colon shortening and promoting histological repair compared to the other treatment groups, suggesting a dose-dependent protective effect of TLYY against UC pathogenesis. These findings are consistent with previous studies reporting that natural medicines alleviate UC by reducing intestinal mucosal injury (Cheng et al., 2023; Liang et al., 2024; Yang et al., 2025). Thus, our data preliminarily confirm that TLYY directly targets colonic inflammatory lesions to attenuate tissue damage.

Inflammatory cytokines and disruption of the intestinal barrier are central to the progression of UC. TNF-α, IL-6, and IL-1β are classic pro-inflammatory cytokines whose excessive release exacerbates intestinal inflammation. Decreased expression of tight junction proteins (ZO-1, Occludin, Claudin-1) facilitates the translocation of endotoxins such as LPS into the bloodstream, further amplifying inflammatory damage (Xiong et al., 2022; Jia et al., 2024). Our results demonstrated that TLYY significantly reduced the levels of TNF-α, IL-6, and IL-1β in the colon tissue of DSS-treated mice, concurrently decreasing serum LPS levels and upregulating the expression of intestinal barrier proteins. These findings indicate that TLYY not only mitigates the inflammatory response by suppressing pro-inflammatory cytokine release but also interrupts the vicious cycle of “intestinal damage-endotoxin translocation-inflammation exacerbation” through the restoration of both physical and chemical intestinal barriers. TLYY is a multi-herbal compound formulation. Extracts from its principal components, such as Pulsatillae Radix, Astragali Radix, and Portulacae Herba, have been shown to alleviate UC-related symptoms by reducing inflammatory injury, exerting antioxidant effects, and enhancing barrier protection (Sahu et al., 2025; Liang et al., 2025; Zhang Y. et al., 2025).

Gut microbiota dysbiosis is a key factor in UC pathogenesis, influencing intestinal physiology and systemic inflammation via the gut-microbiota-host axis. Our results revealed that DSS induction significantly reduced gut microbial diversity in mice, as evidenced by decreased Shannon and Simpson indices-a finding consistent with the characteristic reduction in microbial diversity observed in patients with inflammatory bowel disease (Schult-Hannemann et al., 2026; Wu et al., 2023). Intervention with a high dose of TLYY significantly restored microbial diversity, and PCoA results showed that the microbiota structure in the TLYYH group clustered closer to the NC group, indicating that TLYY effectively reverses DSS-induced gut microbiota dysbiosis. At the phylum level, DSS administration notably increased the Firmicutes/Bacteroidota (F/B) ratio, a shift associated with enhanced intestinal permeability and inflammation (Ma et al., 2025; Li et al., 2024). TLYYH treatment significantly lowered the F/B ratio, correcting this imbalance. At the genus level, TLYYH exerted differential modulatory effects on specific bacterial taxa. TLYY significantly increased the relative abundances of beneficial genera, including *Lactobacillus*, *Akkermansia* and *Odoribacter*. The restoration of *Akkermansia*, a mucin-degrading probiotic critical for mucus layer integrity (Rodrigues et al., 2022; Wang et al., 2024), may directly contribute to barrier repair. *Lactobacillus*, known for producing anti-microbial metabolites and modulating immunity, likely supports a healthier microbial environment. Conversely, TLYYH intervention markedly suppressed potentially harmful genera, such as *Helicobacter* and *Desulfovibrio*. *Helicobacter* infection is associated with chronic gastritis, peptic ulcers, gastric cancer, and gastric lymphoma (Kaliyamoorthy et al., 2025), *Desulfovibrio*, on the other hand, is regarded as a “potential pro-inflammatory” genus. As a Gram-negative anaerobic bacterium, it primarily colonizes the colon, where a key physiological characteristic is its capacity to reduce sulfate to hydrogen sulfide (H2S)-a compound known to exert cytotoxic effects on the intestinal mucosa. The over-proliferation of *Desulfovibrio* has been consistently reported in UC patients (An et al., 2023; Xie et al., 2024). Correlation analysis further strengthened the biological relevance of these microbial shifts. Beneficial genera (*Akkermansia*, *Lactobacillus*) showed significant negative correlations with colonic pro-inflammatory cytokines (TNF-α, IL-1β, IL-6) and serum LPS levels. In contrast, the suppressed harmful genera (*Helicobacter*, *Desulfovibrio*) exhibited significant positive correlations with these inflammatory markers. Collectively, these findings suggest that TLYY may alleviate UC through enriching beneficial microorganisms, suppressing harmful bacteria, and remodeling the overall structure of the gut microbiota.

Excessive oxidative stress is a critical driver of intestinal mucosal damage in UC. The overproduction of reactive oxygen species (ROS) can directly damage cellular components (lipids, proteins, and nucleic acids), thereby exacerbating inflammation and tissue injury (Zhou et al., 2025; Yan et al., 2023). In this study, DSS induction significantly decreased the activities of antioxidant enzymes (SOD and GSH-Px) and increased the level of MDA in both serum and colon tissue, indicating successful establishment of an oxidative stress imbalance. Treatment with TLYYH significantly enhanced SOD and GSH-Px activities and reduced MDA content. Furthermore, immunohistochemical analysis demonstrated that TLYYH markedly up-regulated the protein expression of SOD1 and SOD2 in colon tissue, suggesting that the antioxidant effect of TLYY involves not only enhancing enzymatic activity but also modulating the expression of antioxidant enzymes at the molecular level. A close interrelationship exists between gut microbiota and oxidative stress. Beneficial bacteria modulated by TLYY, such as *Lactobacillus* and *Akkermansia*, have been reported to exhibit strong antioxidant properties (Guo et al., 2024; Zhang T. et al., 2025). Conversely, the amelioration of oxidative stress helps to create a healthier intestinal microenvironment, which in turn favors the proliferation and stability of these beneficial microbes. Correlation analyses in this study further support this interactive relationship, indicating that TLYY alleviates DSS-induced intestinal mucosal injury through a synergistic mechanism involving microbiota regulation and oxidative stress amelioration.

Disturbances in the gut microbiota are often accompanied by alterations in metabolite profiles. Fecal metabolomics directly reflects the metabolic interplay between the host and its microbiota, thereby providing insights into the mechanism of TLYY at the metabolic level. Short-chain fatty acids (SCFAs), key microbial metabolites, are characteristically reduced in UC. Acetate, propionate, and butyrate exert anti-inflammatory effects by modulating mucosal immunity and maintaining intestinal homeostasis (Zhang Y. et al., 2025; Li et al., 2024). In this study, levels of acetic acid, propionic acid, butyric acid, and total SCFAs were significantly decreased in DSS-treated mice. Intervention with a high dose of TLYY (TLYYH) significantly restored their concentrations. Spearman correlation analysis further demonstrated that the abundance of beneficial bacteria such as *Lactobacillus* and *Akkermansia* was positively correlated with SCFA levels, whereas harmful bacteria including *Helicobacter* and *Desulfovibrio* were negatively correlated. These results suggest that TLYY may alleviate UC by promoting beneficial bacteria and enhancing SCFA production.

To further elucidate the synergistic mechanism through which TLYY alleviates UC via “microbiota regulation-metabolic improvement,” we performed untargeted metabolomic analysis on mouse fecal samples. In the DSS group, 125 metabolites were significantly altered (57 in positive and 68 in negative ion mode), implicating 20 core metabolic pathways. TLYYH intervention substantially reversed DSS-induced metabolic disturbances, modulating 109 differential metabolites (49 in positive and 60 in negative ion mode) and some metabolic pathways such as the AGE-RAGE signaling pathway, Starch and sucrose metabolism and Cytochrome P450-mediated xenobiotic metabolism. At the metabolite level, TLYYH significantly downregulated toxic metabolites elevated by DSS, such as geldanamycin and bacilysin, while upregulating beneficial metabolites including dehydroascorbate, pseudobaptigenin, salicylurate, and ganoderic acid A. Among these, our study found that *Akkermansia* was positively correlated with ganoderic acid A and dehydroascorbate. However, *Helicobacter* and *Desulfovibrio* were significantly negatively correlated with pseudobaptigenin. Relevant studies indicate that dehydroascorbate stimulates pentose phosphate pathway enzymes, increases intracellular glutathione levels, and inhibits hydrogen peroxide-induced mitochondrial transmembrane potential dissipation and cell death, highlighting its antioxidant role (Puskas et al., 2000). Ganoderic acid A has been shown to effectively prevent colitis, maintain epithelial and mucus barrier integrity, regulate gut microbiota, and promote tryptophan metabolism (Kou et al., 2024). Collectively, at the metabolic level, TLYY ameliorates the intestinal microenvironment by counteracting DSS-induced metabolic dysregulation, restoring core metabolic pathway functions, and elevating levels of beneficial metabolites such as SCFAs.

## Conclusion

Taoling Yuyang Decoction can improve DSS-induced colitis in mice through multiple mechanisms, including reducing oxidative stress, inhibiting inflammatory response, repairing intestinal barrier function, remodeling intestinal flora homeostasis, restoring SCFAs metabolism, and regulating fecal metabolic pathway. The effect is better at high dose, providing a new candidate for clinical treatment of UC. However, limitations remain in the current research. Future studies should integrate multi-omics technologies such as transcriptomics and proteomics to further elucidate the molecular mechanisms by which TLYY modulates the “gut microbiota-metabolism” axis, thereby advancing integrated traditional Chinese and Western medicine strategies for the treatment of ulcerative colitis.

## Data Availability

The data presented in this study are publicly available. Data are deposited in National Microbiology Data Center (NMDC) with accession numbers: NMDCX0002215 (https://nmdc.cn/resource/attachment/detail/NMDCX0002215) and Bio Project: PRJNA1451097 (https://www.ncbi.nlm.nih.gov/sra/?term=PRJNA1451097).

## Footnotes

1
https://www.biocloud.net

2
https://www.biocloud.net/
