# Supplementary material for: Taoling Yuyang Decoction alleviates DSS-induced ulcerative colitis in mice by regulating gut microbiota -metabolite axis and restore intestinal barrier function
Source: Front Microbiol. 2026 Apr 29;17:1750380. doi: 10.3389/fmicb.2026.1750380 (PMC13167968; doi:10.3389/fmicb.2026.1750380)
Supplement: Supplementary file 1 [file Supplementary_file_1.docx]

Supplementary Material

**Taoling Yuyang Decoction Alleviates DSS-Induced Ulcerative Colitis in Mice by Regulating Gut Microbiota -Metabolite Axis and Restore Intestinal Barrier Function**

Dongyang Liu ^1^, Deshuang Lu ^2^, Ziwen Zhang^2^, Chang Zhang^2^,Shuang Liu^3^, Jinlai Song^4^, Xingrui Cui^5^, Zhuoyue Dong^2^, Lin Li^2^*, Guoying Liang^1,3^* and Chunjing Zhang^2,^*

1. Heilongjiang University Of Chinese Medicine, Harbin 150040,China; ldy19991010@163.com(D.-Y.L.)
2. Department of Medical Technology, Qiqihar Medical University, Qiqihar 161006, China; [ldsytsl1026@163.com](mailto:ldsytsl1026@163.com) (D.-S.L.); [qmuzzw@163.com(Z.-W.Z.);](mailto:qmuzzw@163.com(Z.-W.Z.),) [qqhrzc369@163.com](mailto:qqhrzc369@163.com) (C.Z.); [DSHvina@163.com](mailto:DSHvina@163.com) (Z.-Y.D.); [lilin@qmu.edu.cn(L.L.),](mailto:dalin36@163.com(L.L.),) [000243@qmu.edu.cn (C.-J.Z.)](mailto:cjzhang2005@163.com(C.-J.Z.),)
3. The First Affiliated Hospital of Heilongjiang University of Chinese Medicine, Harbin 150040, China;1326675716@qq.com(S.L.) ;1078720583@qq.com(G.-Y.L.)
4. College of Basic Medicine,Heilongjiang University of Chinese Medicine,Harbin 150040,China; [2744640761@qq.com(J.-L.S.)](mailto:1042790721@qq.com(J.-L.S.))
5. Jiangsu Province Hospital with Integration of Chinese and Western Medicine, Nanjing University of Chinese Medicine, Nanjing 210023,China; 1042790721@qq.com(X.-R.C.)

***** Correspondence: [lilin@qmu.edu.cn(L.L.);](mailto:dalin36@163.com(L.L.),) [1078720583@qq.com(G.-Y.L.);](mailto:1078720583@qq.com(G.-Y.L.);) [000243@qmu.edu.cn (C.-J.Z.)](mailto:cjzhang2005@163.com(C.-J.Z.),); Tel.: Tel.: +86-0452-2663878

## Supplementary Tables

Supplementary Table 1. Chemical composition of TLYY.

| Constituents | m/z | Reference Ion | Formula | Class | Group Area |
| --- | --- | --- | --- | --- | --- |
| Hpro | 116.07061 | [M+H]+ | C5H9NO2 | Carboxylic acids and derivatives | 48436006433 |
| Phosphoric acid | 96.95919 | [M-H]- | H3O4P | Non-metal oxoanionic compounds | 32657558656 |
| (4S)-4-Amino-5-hydroxypentanoic acid | 116.07063 | [M+H]+ | C5H11NO3 | Carboxylic acids and derivatives | 26786526705 |
| Citric acid | 191.01877 | [M-H]- | C6H8O7 | Carboxylic acids and derivatives | 26784691564 |
| isomaltulose | 377.08551 | [M-H]2- | C12H22O11 | Organooxygen compounds | 21710813997 |
| L-Malic acid | 133.01314 | [M-H]- | C4H6O5 | Hydroxy acids and derivatives | 14444618901 |
| L-Arginine | 175.11884 | [M+H]+ | C6H14N4O2 | Carboxylic acids and derivatives | 13973684663 |
| Cefprozil impurity A | 151.03522 | [2M+NH4]+ | C8H9NO3 | Carboxylic acids and derivatives | 13374198319 |
| Gluconic acid | 195.05031 | [M-H]- | C6H12O7 | Organooxygen compounds | 8911621516 |
| 11-(Hydroxymethyl)-2,11-dimethyl-3-oxotricyclo[4.3.2.0~1,5~]undecane-9-carboxylic acid | 231.1378 | [M+H]+ | C15H22O4 | Prenol lipids | 8534482034 |
| 5-Methoxypsoralen | 215.03171 | [M-H]- | C12H8O4 | Coumarins and derivatives | 7843324884 |
| Ganoderic Acid D | 515.30005 | [M+H]+ | C30H42O7 | Prenol lipids | 7657633996 |
| PC(16:0/0:0) | 496.34042 | [M+H]+ | C24H50NO7P | Glycerophospholipids | 7579899650 |
| Guignardone A | 291.15897 | [M+H]+ | C17H22O4 | Prenol lipids | 7388611923 |
| Hirtellanine A | 379.08197 | [M-H]- | C21H16O7 | Isoflavonoids | 7310043737 |
| Pidolic Acid | 130.05 | [M+H]+ | C5H7NO3 | Carboxylic acids and derivatives | 6981802900 |
| (+)-homoproline | 130.08658 | [M+H]+ | C6H11NO2 | Carboxylic acids and derivatives | 6817147344 |
| BETAINE | 118.08613 | [M+H]+ |  | Carboxylic acids and derivatives | 5675966218 |
| 3-O-feruloyl-D-quinic acid | 367.10333 | [M-H]- | C17H20O9 | Organooxygen compounds | 5638274318 |
| QUINIC ACID | 191.05566 | [M-H]- | C7H12O6 | Organooxygen compounds | 5435675466 |
| Guanidinoacetic acid | 118.06506 | [M+H]+ | C3H7N3O2 | Carboxylic acids and derivatives | 4973806950 |
| Monoisobutyl phthalic acid | 149.02333 | [M+H]+ | C12H14O4 | Benzene and substituted derivatives | 4852561440 |
| Lactate | 89.02319 | [M-H]- | C3H6O3 | Hydroxy acids and derivatives | 4766499232 |
| L-Isoleucine | 132.10175 | [M+H]+ | C6H13NO2 | Carboxylic acids and derivatives | 4745407556 |
| Germacrone | 219.17372 | [2M+H]+ | C15H22O | Prenol lipids | 4619499976 |
| TREHALOSE | 341.10934 | [M-H]- | C12H22O11 | Organooxygen compounds | 4472930270 |
| 11-chloro-12-hydroxy-octadecanoic acid | 333.22845 | [M-H]- | C18H35ClO3 | Fatty Acyls | 4407356566 |
| L-Asparagine | 133.06073 | [M+H]+ | C4H8N2O3 | Carboxylic acids and derivatives | 3915480534 |
| Linustatin | 248.11217 | [M+Na]+ | C16H27NO11 | Organooxygen compounds | 3475099782 |
| 3-Feruloylquinic acid | 367.10339 | [M-H]- | C17H20O9 | Organooxygen compounds | 3450311216 |
| 5,7-dihydroxy-4H-chromen-4-one-3-O-beta-D-xylopyranoside | 325.05655 | [M-H]- | C14H14O9 | Benzopyrans | 3388589379 |
| (9Z)-5,8,11-Trihydroxy-9-octadecenoic acid | 329.23279 | [M-H]- | C18H34O5 | Fatty Acyls | 3371200318 |
| Fast Red Base DB-30 | 128.01923 | [M+ACN+H]+ | C7H7ClN2O | Benzene and substituted derivatives | 3070456382 |
| l-canavanine | 177.09808 | [M+NH4]+ | C6H13N3O3 | Carboxylic acids and derivatives | 2967103309 |
| 3,13-dihydroxy-4,6a,6b,8a,11,12,14b-heptamethyl-9,14-dioxo-2,3,4a,5,6,7,8,10,11,12,12a,14a-dodecahydro-1h-picene-4-carboxylic acid | 501.3212 | [M+H]+ | C30H44O6 | Prenol lipids | 2937564240 |
| Erythronolactone | 117.01831 | [M-H]- | C4H6O4 | Lactones | 2853577145 |
| 5-carbamimidamido-2-{[(2,3,4,5-tetrahydroxyoxan-2-yl)methyl]amino}pentanoic acid | 337.17102 | [M+H]+ | C12H24N4O7 | Carboxylic acids and derivatives | 2785134817 |
| Octyl hydrogen phthalate | 279.15881 | [M+H]+ | C16H22O4 | Benzene and substituted derivatives | 2742992830 |
| 1-Oleoyl-sn-glycero-3-phosphocholine | 522.3562 | [M+H]+ | C26H52NO7P | Glycerophospholipids | 2573946310 |
| isocitric acid | 111.00777 | [M-H]- | C6H8O7 | Carboxylic acids and derivatives | 2436521932 |
| Benzal chloride | 158.97765 | [M-H]- | C7H6Cl2 | Benzene and substituted derivatives | 2190651621 |
| Isomaltose | 387.11435 | [M-H]- | C12H22O11 | Organooxygen compounds | 2143972951 |
| D-(+)-Sorbose | 127.03885 | [M+H]+ | C6H12O6 | Organooxygen compounds | 2140690572 |
| Thr-Tyr | 283.13986 | [M+H]+ | C13H18N2O5 | Carboxylic acids and derivatives | 2074583458 |
| Ribonolactone | 165.03964 | [M-H]- | C5H8O5 | Organooxygen compounds | 2042779192 |
| 3-(Carboxymethyl)benzoic acid | 163.03899 | [M+H]+ | C9H8O4 | Benzene and substituted derivatives | 1865278815 |
| Wilforlide A | 455.35181 | [M+NH4]+ | C30H46O3 | Prenol lipids | 1856591648 |
| (2r)-2-(hydroxymethyl)-2-{[(2s,3r,4s,5s,6r)-3,4,5-trihydroxy-6-(hydroxymethyl)oxan-2-yl]oxy}butanenitrile | 278.12286 | [M+H]+ | C11H19NO7 | Organooxygen compounds | 1832176686 |
| N-trans-feruloyl-3-methoxytyramine-4'-O-beta-D-glucopyranoside | 506.20312 | [M+H]+ | C25H31NO10 | Organooxygen compounds | 1753749247 |
| L-TYROSINE | 182.08086 | [M+H]+ | C9H11NO3 | Carboxylic acids and derivatives | 1676426837 |

Supplementary Table 2. Primer sequence

| Primer name | Primer sequence5′-3′ | |
| --- | --- | --- |
| ZO-1 | F | GATGTTTATGCGGACGGTGG |
|  | R | CATTGCTGTGCTCTTAGCGG |
| -Claudin-1 | F | AGGTCTGGCGACATTAGTGG |
|  | R | GGCCAAATTCATACCTGGCA |
| Oclludin | F | CTTAGGCGACAGCGGTGGAG |
|  | R | CCAAGATAAGCGAACCTGCCGA |
| β-actin | F | ACTGTCGAGTCGCGTCCA |
|  | R | ATCCATGGCGAACTGGTGG |

## Supplementary Figures


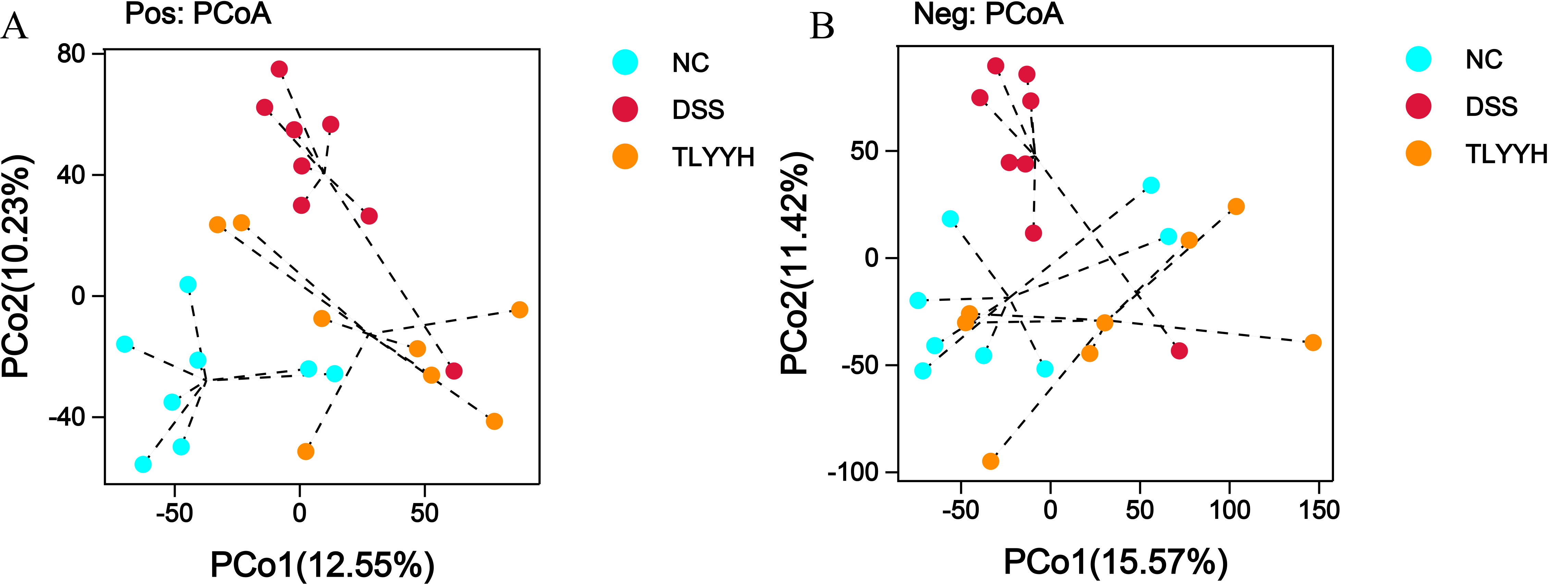


**Supplementary Figure 1.** Principal Coordinate Analysis（PCoA）based on Bray−Curtis distance, (A) Positive ion mode, (B) Negative ion mode.


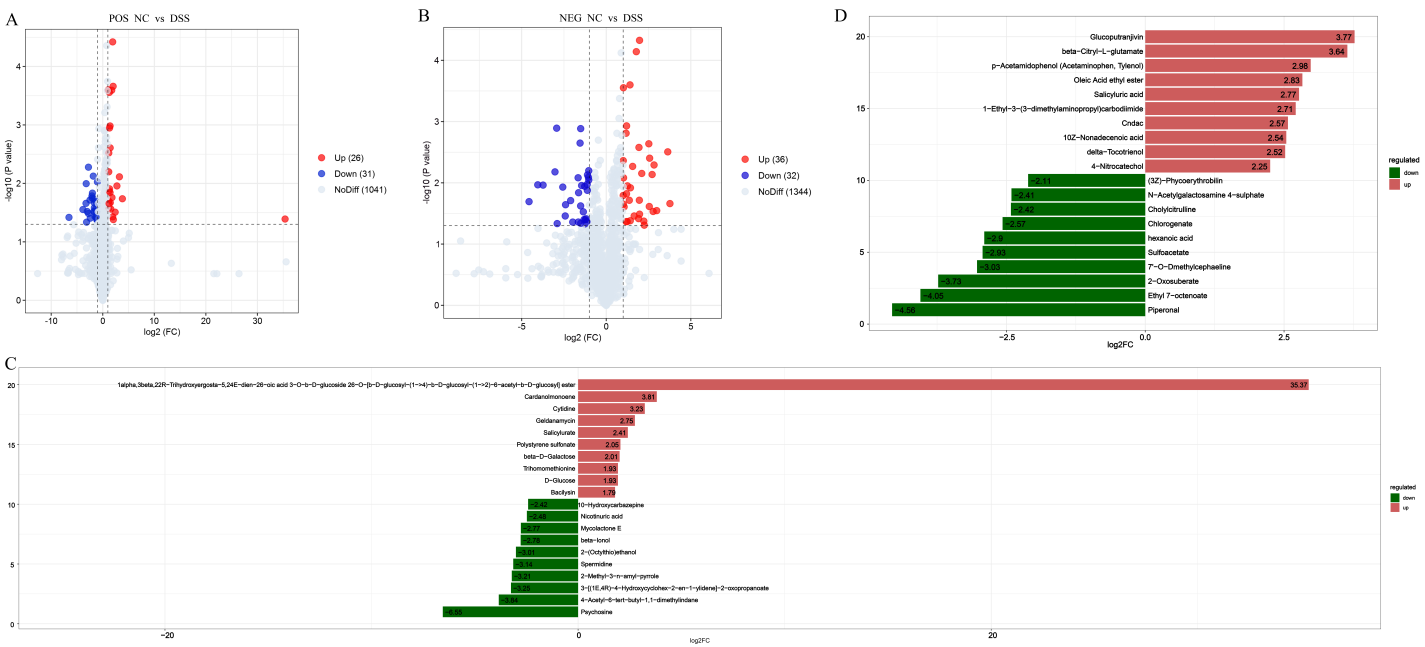


**Supplementary Figure 2.** The fecal metabolites altered in mice. Differential metabolites in fecal identification between NC and DSS in (A, C) positive and (B, D) negative ion modes.(A−B) volcano plot,(C-D) Fold-Change bar chart.
